# Molecular system for an exponentially fast growing programmable synthetic polymer

**DOI:** 10.1038/s41598-023-35720-5

**Published:** 2023-07-12

**Authors:** Nadine Dabby, Alan Barr, Ho-Lin Chen

**Affiliations:** 1 California Institute of Technology, Pasadena, USA; 2 National Taiwan University, Taipei City, Taiwan

**Keywords:** Computer science, DNA computing, DNA nanomachines, DNA nanostructures

## Abstract

In this paper, we demonstrate a molecular system for the first *active* self-assembly *linear* DNA polymer that exhibits programmable molecular exponential growth in real time, also the first to implement “internal” parallel insertion that does not rely on adding successive layers to “external” edges for growth. Approaches like this can produce enhanced exponential growth behavior that is less limited by volume and external surface interference, for an early step toward efficiently building two and three dimensional shapes in logarithmic time. We experimentally demonstrate the division of these polymers via the addition of a single DNA complex that competes with the insertion mechanism and results in the exponential growth of a population of polymers per unit time. In the supplementary material, we note that an “extension” beyond conventional Turing machine theory is needed to theoretically analyze exponential growth itself in programmable physical systems. Sequential physical Turing Machines that run a roughly constant number of Turing steps per unit time cannot achieve an exponential growth of structure per time. In contrast, the “active” self-assembly model in this paper, computationally equivalent to a Push-Down Automaton, is exponentially fast when implemented in molecules, but is *taxonomically less powerful* than a Turing machine. In this sense, a physical Push-Down Automaton can be *more powerful* than a sequential physical Turing Machine, even though the Turing Machine can compute any computable function. A need for an “extended” computational/physical theory arises, described in the supplementary material section S1.

## Introduction

Molecular programming, nanotechnology and synthetic biology raise the prospect of bottom-up fabrication, the manufacture of complex devices that assemble themselves from simpler components. Biological systems fabricate structures with enormous scale and complex behaviors, defined at atomic-scale resolution, which can grow quickly with small programs relative to their object size and algorithmic complexity^1^. A goal in the molecular synthesis field is to build biophysical systems with great complexity and power, with applications to medicine, the environment, and green manufacturing.

In natural biological systems, periods of programmed exponential growth per unit time is common, and perhaps is almost ubiquitous. Understanding and controlling exponential growth will become key, to obtain acceptable reaction yield and performance for practical applications of bottom-up self-fabrication.

Over the past several years, new directions in research have been translating computational algorithms into and out of molecular systems using DNA and other molecular substrates. DNA has been used to build autonomous walkers^2–10^, logic and catalytic circuits^8,11–13^, and triggered assembly of linear^14,15^ and dendritic structures^8^.

**Figure 1:**
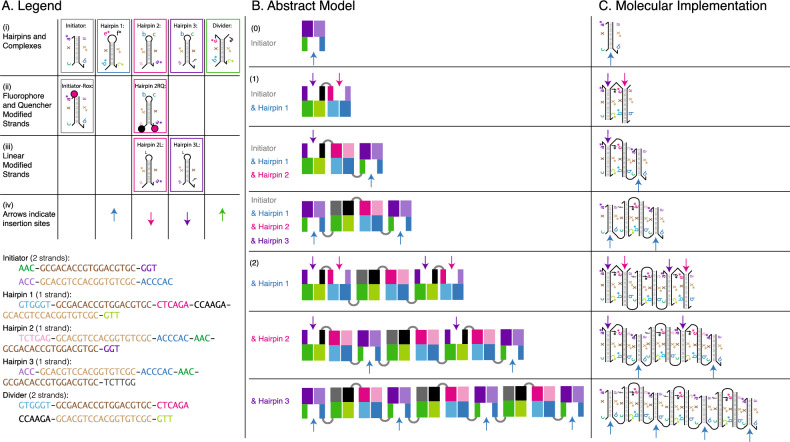
Schematic of our insertional polymer implementation using DNA. The insertional polymer implementation shows the first two rounds of growth. (**A**) Legend shows (i) schematics of the Initiator complex, Hairpin 1, Hairpin 2, Hairpin 3, and Divider complex with sequences color-coded by domain below. Each oligonucleotide is shown with color-coded motifs that correspond to the DNA subsequences below. (ii) The Initiator-ROX complex is a modified Initiator complex with a single fluorophore tag for gel electrophoresis experiments. Hairpin 2RQ is a modified Hairpin 2 molecule with a quencher and fluorophore pair on opposite ends of the molecule, used in the spectrofluorimetry experiments. (iii) Hairpin 2L and Hairpin 3L are inactivated versions of Hairpins 2 and 3, in which the loops are replaced with an inactive poly-T sequence. The color of the boxes around each oligonucleotide in (i) correspond to the insertion arrows in (iv) as follows: a blue arrow indicates an insertion site for Hairpin 1, a pink arrow indicates an insertion site for Hairpin 2, Hairpin 2RQ or Hairpin 2L, a purple arrow indicates an insertion site for Hairpin 3 or Hairpin 3L, a green arrow indicates an insertion site for the Divide complex. (**B**) The abstract model of our system and (**C**) the molecular implementation of our polymer display exponential growth occurs as follows: (0) The Initiator has one insertion site for Hairpin 1 (blue arrow). Insertion of Hairpin 1 is driven forward by the hybridization of 6 new base pairs. (1) After Hairpin 1 inserts into the Initiator, two new insertion sites are generated: one for Hairpin 2 (pink arrow) and one for Hairpin 3 (purple arrow). Hairpin 2 and Hairpin 3 are sequentially inserted (in solution insertion occurs asynchronously), each one generates a new insertion site for Hairpin 1 (blue arrows). After the first round of insertion, two insertion sites for Hairpin 1 are generated from what was initially (in round (0)) one site. (2) A second round of insertion is illustrated. The 4-way branch migration mechanism used in the insertion process is demonstrated in Fig. S2 in the supplementary material.

**Figure 2:**
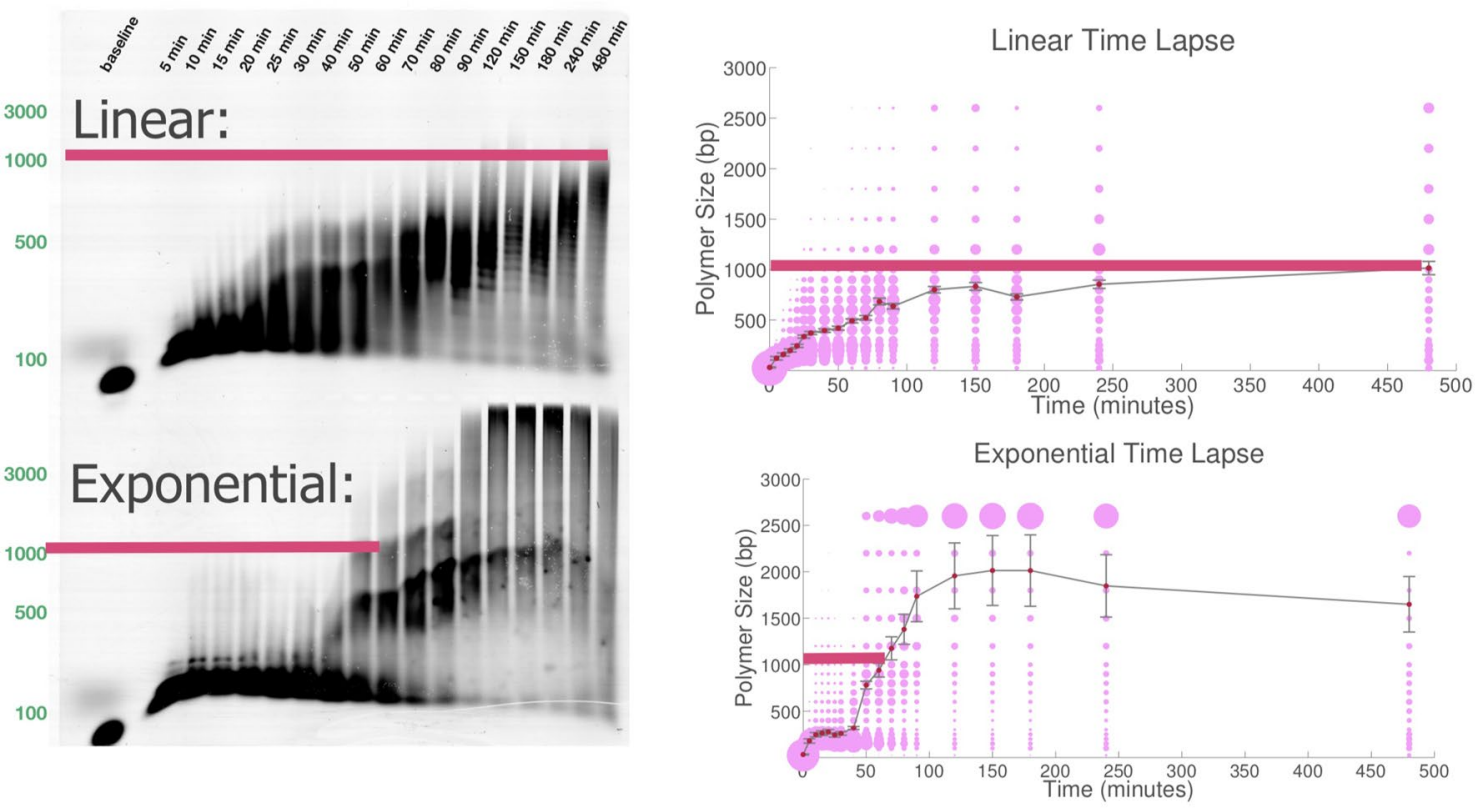
Gel time-lapse studies of linear and exponential polymer growth. The bottom edges of the thick horizontal red lines indicate 1000 base pairs. Top left: Gel time-lapse studies of linear polymer growth. Note that it takes 480 min to reach nearly 1000 base pairs. Super Fine Resolution Agarose non-denaturing gels of the product of a polymerization reaction with 80 nM ROX-labeled Initiator, 1.5 $\mu$M Hairpin 1, and 1 $\mu$M of Hairpin 2 and Hairpin 3L. ROX fluorescence was imaged prior to staining with SYBR Gold. (The SYBR Gold stained gel can be found in Fig. S12). A more complete analysis of this gel was precluded due to the interference of the fluorescent loading dye bromophenol blue as discussed in section "Time Lapse experiments". Top right: Average length of the polymers versus time in the linear system. The size of the purple dots represent the total molecular weight of molecules with that size. Bottom left: Gel time-lapse studies of exponential polymer growth. It takes less than 60 min to reach 1000 base pairs. Super Fine Resolution Agarose non-denaturing gels of the product of a polymerization reaction with 80 nM ROX-labeled Initiator, 1.5 $\mu$M Hairpin 1, and 1 $\mu$M of Hairpin 2 and Hairpin 3. ROX fluorescence was imaged prior to staining with SYBR Gold. (The SYBR Gold stained gel can be found in Fig. S13). Three additional experimental runs of this experiment can be found in Figs. S10, S11 and S14. A more complete analysis of this gel was precluded due to the interference of the fluorescent loading dye bromophenol blue as discussed in section "Time Lapse experiments". Bottom right: Average length of the polymers versus time in the exponential system. Note the rapid increase on the growth rate after the 30-min point which indicates that the growth is nonlinear on the bottom right. Also note from the time labels that the time scale of the columns of the gel and graphs on the left and right do not match, which makes the thick red 1000-base-pair line appear to be longer in the graph on the bottom left and shorter on the bottom right, even though each red line in the bottom two graphs is still around 60 min long. The 1000-base-pair time for the bottom “exponential” graphs, 60 min, is much shorter than the time for the 1000-base-pair red line in the top two “linear” graphs, which is around 480 min long.

The primary task in this paper is to build an exponentially quickly growing molecular assembly in the physical world. We present a programmable molecular model and a molecular implementation of the first *active* synthetic *linear polymer* system that can grow exponentially quickly in Real Time (see Fig. 2). Our molecular system is *not*, however, the first exponentially fast growing structure ever synthesized. Yin et al. constructed a *binary molecular tree* out of DNA^8^. Our system is implemented with DNA and is also capable of a second behavior—splitting or division of polymers. By encoding the order of the nucleotides in the DNA sequence, we can control the interaction of DNA strands, which is how the system is programmed. Our molecular construction (Fig. 1) is inspired by the Hybridization Chain Reaction (HCR) system developed by Dirks and Pierce^14^. The molecular DNA system is computationally equivalent to a Pushdown Automaton^16,17^.

The molecular insertion system we designed, computationally equivalent to a Pushdown Automaton, when implemented in molecules as described in Fig. 1C, could perform exponential growth tasks that some Turing-Complete molecular systems could *not* perform, such as the DNA Tile Assembly Model^18^, even though Turing Complete systems can in principle, implement any computable function. The Turing-Complete DNA Tile system cannot achieve “exponentially quick” growth in real physical time, while the Pushdown Automaton in Fig. 1C can, even when both are implemented molecularly. Yet the Turing-Complete molecular system can, in theory, compute “anything.”

This was surprising to us at first, since a Turing-Complete system is taxonomically more powerful, in a purely computational sense, than a Push-Down Automaton, which is *not* Turing complete.

The observation led us to have a discussion in the supplementary material section S1 to identify a need for a new type of combined physical/computational theory for “Extended Physical Computation.” This would aid the understanding of *programmable physical systems*, especially in the context of exponential growth. It also relates to the the body of work initiated in the 1980’s by Carver Mead, John Hopfield and Richard Feynman, on the Physics of Computation^19,20^. A new type of theoretical and conceptual framework could be useful for understanding how to build and analyze exponentially growing, complex, programmable physical systems for the technology of bottom-up self-fabrication.

The remainder of this paper is organized as follows: In section "Schematic and computational model for “active” self-assembly" we describe the formal Push-Down Automaton computational model we use for our active insertion method for self-assembly. In section "Molecular implementation", we present experimental methods that describe our molecular implementation. In section "Exponential growth results", we present the exponential growth results, as well as the exponential growth mechanism controls and the kinetics of parallel insertion, and time lapse experiments. In section "Methods to generate other behaviors", we describe methods to generate other behaviors, such as division and treadmilling. We end the paper in section "Conclusions" with conclusions, and then present a Supplementary Section containing additional materials for the experimental methods.

## Schematic and computational model for “active” self-assembly

In this section we define the first molecularly implementable active self-assembly model (shown in Fig. 1). We introduce a theoretical framework for provably knowing what actions, behaviors, and life-like qualities can emerge from a given set of simple modular units. We will use some of the theoretical approaches that computer science has for determining the complexity and difficulty of solving computational problems. Our approach arises out of the fact that molecules do certain things well and other things badly, and digital computers do other types of things well and badly.

As a starting point we note that the abstract Tile Assembly Model (aTAM)^18^ is a “passive” self-assembly system that formally couples computation with shape construction. It is a computational model that can be directly implemented in DNA molecules^21–23^. Implements aTAM systems, which assemble self-similar fractals, counters, and digital circuits using DNA molecules. Winfree showed that the tiles are capable of universal computation^18^. Such a system is said to be “Turing-complete”. Because the Tile Assembly Model is Turing-complete, it is capable of computing anything that another computer can compute with at most a polynomial time slowdown, but it cannot compute any arbitrary task. There are many behaviors that are not “computations” in a classical sense. Examples include exponential growth, and molecular motion relative to a surface. The tiles cannot implement these behaviors because (a) there is no instruction for moving or rotating a tile relative to a surface and (b) passive self-assembly is exponentially slower than active self assembly. Several extensions of the aTAM have been proposed, such as staged (two-handed, hierarchical) assembly^24–27^ and signal passing tiles^28^ have been proposed. These systems are more powerful than the aTAM (signal passing tiles are more fuel-efficient in simulating Turing machines^28^; staged assembly system can perform superlinear growth^26^. However, these extensions are still passive and cannot achieve the abovementioned behaviors. The theoretical nubot model^29^ is not molecularly implementable today, but it could achieve exponential growth in theory. The behaviors that we catalogued are different from typical computations because they demonstrate a notion of physicality that is not captured by traditional computational theory. These behaviors are in a class that we call “programmable behaviors”. In order to achieve these behaviors, a tile system requires the logic for how it will grow and move, and the speed conferred by allowing individual molecules to change the existing structure, but the system does not need to be Turing-complete.

As we will demonstrate, a sufficiently expressive implementation of an “active” molecular self-assembly approach can achieve these behaviors. We derive a new type of “active” self-assembly system that can be formally defined and easily implemented in molecules.

Molecular biology needs a better theoretical framework for understanding the complexity of subsets of molecules that interact with each other to generate behaviors. Computer Science has such a framework but it deals with computational complexity—thus we can say how “hard” a particular mathematical problem is by analyzing how much time and space a computer requires to solve it. On the other hand, in other parts of Biology, we can’t say how computationally “hard” it is to generate behaviors like metamorphosis (the changing of one shape into another) or treadmilling (the growth of a linear polymer in one direction while it shrinks in the other direction). The absence of this ability to distinguish how hard it is to produce a desired behavior in a molecular system is a limitation in the fields of synthetic chemistry and biology.

In the absence of biological measures of complexity we map our system onto a computational framework, by proving theorems regarding the “expressive power” of the model we define. The expressive power of our system is equivalent to context-free grammars^16,17^. This system is capable of implementing exponential growth and can construct fixed-length linear polymers in poly-logarithmic time^16,17,30^.

### Formal molecular model description

In our model, each construction begins with an initiator, and grows via the insertion of simple units that we call monomers (Fig. 1). We assume that each type of monomer in the system is present in infinite amounts. Monomers can be inserted into the middle of the structure and increase the length of the structure.

The detailed description of initiators, monomers, and the insertion rules follows:

1. We have two finite sets of symbols $\Gamma \ =\ \{a_1, a_2, a_3, a_4, \dots \}$ and $\Gamma ^*\ =\ \{a_1^*, a_2^*, a_3^*, a_4^*, \dots \}$. Each pair $a_i$ and $a_i^*$ are called *complementary* to each other.
2. There are *k* monomers, each is described by a quadruple of symbols (*a*, *b*, *c*, *d*) and either a plus sign or a minus sign. The plus and minus sign indicate the directionality of the molecules and are used in mapping the model onto a direct DNA implementation, which requires both $5^{\prime }$ and $3^{\prime }$ sequences. (For example, $(a_4, a_7, a_6^*, a_1)+$ or $(a_5, a_7, a_2^*, a_3^*)-$.) Each monomer has a concentration *c*. We assume that the total concentration is at most 1. For example, in the exponential growing system described in Fig. 1, Hairpin 1 can be described as $(b^*, e^*, f^*, c^*)+$ and Hairpin 2 can be described as $(c, a^*, e, b)-$.
  Notice that all hairpin sequences are represented in a clockwise ordering, but the ones marked with a “+” and the ones marked with a “−” begin at different locations on the hairpin. The hairpins marked with a “+” begin at one of the toeholds and the hairpins marked with a “−” begin in the middle of the hairpin loop. The insertion of a “+” hairpin generates insertion sites where only “−” hairpins can insert, and vice versa, but the choice of “+” and “−” can be arbitrary.
3. The initial state can be described by two pairs of symbols (*a*, *b*), (*c*, *d*). Either *a* and *d* are complementary to each other or *b* and *c* are complementary to each other. Each of these pairs is considered a monomer.
4. An *insertion site* can only exist between two consecutive monomers: e.g., in the initial state (*a*, *b*) and (*c*, *d*) belong to two different monomers. For example, in Fig. 1, the initial state (Initiator) can be described as $(a, b), (c, a^*)$ forming an insertion site.
5. Only the following insertion rules are possible:
  - If there are two consecutive monomers connected in the structure such that the first one ends with the pair $(e, a^*)$ and the second one starts with the pair $(d^*, f)$, where *e* and *f* are complementary with each other, then any monomer of the form $(a,b,c,d)+$ can insert between those two groups, and add a group of symbols (*a*, *b*, *c*, *d*) in the middle. $(e, a^*), (d^*, f)$ is called an *insertion site*.
  - If there are two consecutive monomers connected in the structure such that the first one ends with $(d^*, e)$ and the second one starts with $(f, a^*)$, where *e* and *f* are complementary with each other, then any monomer of the form $(a,b,c,d)-$ can insert between these two groups and add a group of symbols (*c*, *d*, *a*, *b*) in the middle. $(d^*, e), (f, a^*)$ is called an *insertion site*.
6. If a particular insertion is applicable, it occurs at time *x*, where *x* is an exponential random variable with rate *c*, where *c* is the concentration of the monomer inserted.
7. A *polymer* is a sequence of tuples of symbols reachable from the initial state, where the first and last tuples are pairs of symbols and the middle tuples are monomers (as defined in rule 2). A *terminal polymer* is a polymer such that no monomers exist in the system that can be inserted at any of the insertion sites available on that polymer. The *length* of the polymer is defined as the number of monomers that it contains. For example, in Fig. 1, Hairpin 1, $(b^*, e^*, f^*, c^*)+$, can insert into the Initiator, which implements the initial state $(a, b), (c, a^*)$ to form a new polymer $(a, b), (b^*, e^*, f^*, c^*), (c, a^*)$ with two new insertion sites $(a, b), (b^*, e^*)$ and $(f^*, c^*), (c, a^*)$. Notice that the sequences *a* and $a^*$ were bound together before the insertion and are separated after the insertion. After that, Hairpin 2, $(c, a^*, e, b)-$ can insert into the first new insertion site $(a, b), (b^*, e^*)$ to form the structure $(a, b), (c, a^*, e, b), (b^*, e^*)$, generating another new insertion site $(a, b), (c, a^*)$. Hairpin 3 can also insert into the second insertion site $(f^*, c^*), (c, a^*)$ and generate the same insertion site $(a, b), (c, a^*)$.

How do we tell if a polymer is terminal? The only way is to check for the condition, at each site, whether there exists a monomer that can insert according to the insertion rules listed in 5(a) and 5(b). If no monomers can insert, then the polymer is terminal.

## Molecular implementation

Given any system described above, there is a direct implementation of monomers into a set of DNA molecules. By encoding the order of the nucleotides in a DNA sequence, we can control the interaction of DNA strands. Subsequences of these strands are called domains and it is their binding (hybridization) and unbinding (disassociation) from complementary domains that determines what a system can do. In DNA nanotechnology, dynamic systems of DNA molecules can be controlled by toeholds, the short sequences of DNA that are complementary to single stranded domains in a target molecule^31,32^. Toeholds serve as the inputs to dynamic DNA systems and initiate branch migration processes, the random walk process of bond breaking and formation that results in the exchange of one strand in the duplex for another single strand with the same sequence.

Any system described in our model can be implemented by designing DNA hairpins and an initiator complex as follows:

For every monomer $(a,b,c,d)-$, we add a hairpin with domains $(a, x, b, c, x^*, d)$, where *x* (composed of 18 bases) is the long stem of the hairpin. For every monomer $(a,b,c,d)+$, we add a hairpin with domains $(a, x^*, b, c, x, d)$. The initiator is $(a, x^*, b)$ binding with (*c*, *x*, *d*). The insertion rules defined in the model correspond to all possible reactions that can happen in the corresponding molecular system.

In addition to the monomer $(a,b,c,d)+$ (or minus), we can also have a new type of monomer $(a,b)(c,d)+$ that we call a divider monomer. The reaction available for $(a,b)(c,d)+$ is exactly the same as that for $(a,b,c,d)+$, except that after $(a,b)(c,d)+$ inserts, the polymer will be cut between (*a*, *b*) and (*c*, *d*) and divided into two parts , as will be described in section "Division".

Figure 1C shows the molecular implementation of our exponential growth system. Hairpin 1 (H1) and the Initator (I) react first, this results in two new insertion sites: one that is complementary to Hairpin 2 (H2), and another that is complementary to Hairpin 3 (H3). Upon insertion of H2 and H3 into the growing polymer, two new insertion sites that are complementary to H1 are generated. Thus for every initial H1 insertion site, each round of insertions creates two new H1 insertion sites.

The initial reaction (insertion of H1 into the Initiator complex) is driven by the hybridization of six new base pairs. After that, each new hairpin that is inserted adds nine base pairs to the system. Some of these steps become reversible as the system approaches equilibrium. The free energy and reversibility of toehold-mediated four-way branch migration is explored in depth in^33^. Other design lengths and sequences were explored (Tables S1, S2, S3, S4), but these resulted in a larger system leak (an undesired molecular interaction) than the sequences presented here (see Figs. S3, S24, S25, S26 and the discussion in section "Treadmilling").

We created a new molecular system that grows linearly, which we compared to the exponential growth system. To achieve this, we used the same monomers as in the exponential growth system, except that we replaced Hairpin 3 with an inactive version called Hairpin 3L. Hairpin 3L contains a poly-T sequence instead of the loops that normally create a new insertion site for Hairpin 1 after Hairpin 3 inserts. As a result, when Hairpins 1, 2, and 3 are inserted, they generate only one new insertion site for Hairpin 1 to bind to, instead of the two insertion sites created by the exponential growth system. This limits the system’s growth to be linear instead of exponential.

In addition to the insertional monomers that grow the polymer, we introduce a new type of monomer, which we call a Divide complex, that upon insertion splits the polymer into two pieces. This tool can be used to generate a population of polymers in exponential time (Section "Division").

Figure 1A is a legend for the set of DNA molecules used in this section. Each oligonucleotide complex (Initiator, Hairpin 1, Hairpin 2, Hairpin 3, and Divide) is shown with color-coded motifs (purple, green, blue, brown, pink, and black) that correspond to the colored DNA subsequences (Fig. 1A; see Table S1 for all sequences). The Initiator-ROX complex is a modified Initiator complex with a single fluorophore tag for gel electrophoresis experiments. Hairpin 2RQ (H2RQ) is a modified Hairpin 2 molecule with a quencher and fluorophore pair on opposite ends of the molecule, used in the spectrofluorimetry experiments. Hairpin 2L (H2L) and Hairpin 3L (H3L) are inactivated versions of Hairpins 2 and 3, in which the loops are replaced with a poly-T sequence. The boxes around each oligonucleotide correspond to the insertion arrows as follows: a blue arrow indicates an insertion site for Hairpin 1, a pink arrow indicates an insertion site for Hairpin 2, Hairpin 2RQ or Hairpin 2L, a purple arrow indicates an insertion site for Hairpin 3 or Hairpin 3L, and a green arrow indicates an insertion site for the Divide complex (Fig. 4A).

In each diagram, we utilize a domain abstraction for referring to stretches of consecutive nucleotides that act as a unit in binding to complementary stretches of nucleotides. Domains are represented by Latin letters (Fig. 1). Letters followed by an asterisk denote complementary domains, e.g.: **x** is complementary to x*. Single-stranded molecules of DNA (henceforth strands) are comprised of concatenated domains. DNA complexes are composed of two or more noncovalently-bound strands. There are two types of toeholds in our system: long toeholds that indicate a stronger desired interaction (six bases in length) and short toeholds that indicate a weaker desired interaction (three bases in length).

## Exponential growth results

We confirm exponential growth by measuring the conversion of monomers into a product. We then qualitatively measure the size of products over time. Finally, we verify the predicted structure using Atomic Force Microscopy.

### Exponential growth mechanism controls

We tested each insertion step in the exponential growth mechanism by using the inactivated versions of of Hairpins 2 and 3 in which the binding loop is replaced by an inactive sequence of nucleotides (Fig. S3). Hairpin 2L and Hairpin 3L were added to the Initiator and Hairpin 1 both individually (this results in exactly one insertion event) and together with the normal version of the other hairpin (i.e.: Hairpin 2L and Hairpin 3), which results in linear growth. We note that there is more product in lanes 14 (I, H1, H3L) and 15 (I, H1, H3) than there is in lanes 12 (I, H1, H2L) and 13 (I, H1, H2).

The reactants in lanes 12 and 14 can only proceed through two steps of the polymerization reaction due to the inactivated strands. At equilibrium (after 6 hours) there is more dimerization between the Initiator-Hairpin 1 complex and Hairpin 3L than there is between the Initiator-Hairpin 1 complex and Hairpin 2L. Thus Hairpin 3 appears to have a greater affinity to the Initiator-Hairpin 1 complex than Hairpin 2. This observation implies that the two reactions have different rate constants, Hairpin 2 is either slower to react with its insertion site or faster to dissociate from its insertion site than Hairpin 3 (or both).

The reader may observe the presence of faint extra bands in the lanes that contain only individual hairpins. These are dimerized hairpins that form in small amounts from individual hairpins when the strands are annealed. We minimize their presence by snap cooling. Snap cooling the hairpins results in the same amount of dimerized monomers as gel purification (data not shown). All hairpins except for the Initiator were snap cooled prior to experiments. The Initiator is a gel-purified duplex composed of two molecules of DNA.

In order to ensure that polymers were not randomly joining each other over time, we observed the linear systems (I, H1, H2) and (I, H1, H3) and the exponential system (I, H1, H2, H3) when all molecules were added at the same concentration. If the polymers were randomly joining at the ends, we would see a shift in the length of the polymers over time. Figure S4 shows that there is minimal joining as the polymer bands do not shift upward over time.

### The kinetics of parallel insertion

We examined the kinetics of the conversion of monomers into the polymer by adding a fluorophore and quencher pair to the opposite ends of Hairpin 2. Before reaction, the fluorophore is quenched. Upon incorporation of the hairpin into the DNA polymer, the quencher and fluorophore pair are separated, and the fluorescence of the solution increases (Fig. S5).

**Figure 3:**
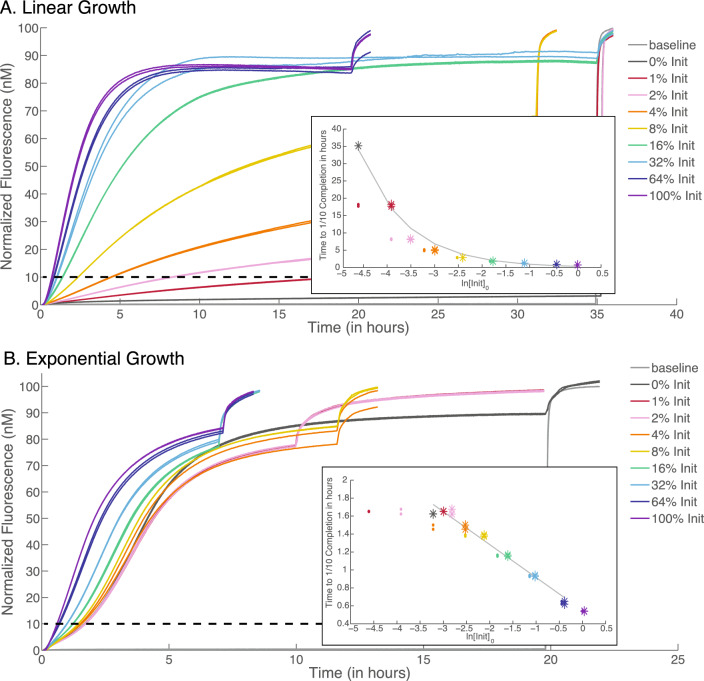
Polymer growth kinetics examined via fluorescence. The legend on the right shows different concentrations of the Initiator with a color code, where $1\%$ is 1 nM. (**A**) Linear polymer growth kinetics are observed in a fluorescence time course when inactivated Hairpin 3L is substituted for Hairpin 3. As Hairpin 2RQ is incorporated into the growing polymer, the system’s fluorescence increases: this illustrates the conversion of hairpins into polymers (all hairpins are present at 100 nM) with varying amounts of Initiator. Inset: Graph of the linear fit of the time required to reach the $10\%$ (of the 100 nM) completion point as a function of the relative concentration of Initiator to hairpins ($t_{1/10} = 2.3*(0.1*[H1]_0)/([I]_0+0.01)$). The *y*-values in the inset graphs correspond to the intersections between the dashed line to the left ($10\%$ completion) and the curves with corresponding colors. The intervals on the *x*-axis in the inset graphs depend on the amount of modeled leak in the system. Small colored dots correspond to an insertion system where we assume no leak. Large colored asterisks indicate the same points but assume a system leak equivalent to $1\%$ of the Initiator concentration. (**B**) Exponential polymer growth kinetics examined via fluorescence. As Hairpin 2RQ is incorporated into the growing polymer, the system’s fluorescence increases; this illustrates the conversion of hairpins into polymer (all hairpins are present at 100 nM) with varying amounts of Initiator. Inset: Graph of the linear fit of the time required to reach the $10\%$ completion point as a function of the relative concentration of Initiator to hairpins ($t_{1/10} = 0.3667*ln(0.1*[H1]_0/([I]_0 + 0.04)) + 1.245$). The *y*-values correspond to the intersections between the dashed lines to the left ($10\%$ completion) and the curves with corresponding colors. The intervals on the *x*-axis depend on the amount of leak in the system. Small colored dots correspond to an insertion system where we assume no leak. Large colored asterisks indicate the same points but assume a system leak equivalent to $4 \%$ of the Initiator concentration^8^. The insets have the same scale for the x-axes to allow better comparison, but the larger plots have different scales in the x-axes to show the exponential growth more clearly. The scale of the x-axis of the top plot is adjusted to show the final completion level within the width of the figure.

We probed both the linear and exponential polymerization over eight different Initiator concentration values. The time course of fluorescence intensity confirmed linear conversion of hairpins in the system with one inactivated strand (Fig. 3A), and exponential conversion of hairpins in the full system (Fig. 3B).

In order to derive both the linear and exponential growth equations, we made the approximation that the hairpin concentrations remain constant until 10% of the monomers are consumed as in^8^. For more details see Supplementary Section S3.

In a linear growth system, the total mass of polymer product, *P*, grows as a function of initial Initiator concentration, $I_0$, and time, *t*, as follows:

1 $$\begin{aligned} P&= k (I_0 + I_{leak}) t . \end{aligned}$$

The time at which $10\%$ of monomers are consumed, $t_{10\%}$, is

2 $$\begin{aligned} t_{10\%}&= \frac{P_{10\%}}{k (I_0 + I_{leak})}. \end{aligned}$$

Thus, in a linear growth system, the time to $10\%$ completion of polymer growth ($10\%$ conversion of hairpins) is inversely proportional to initial Initiator concentration. When plotted on a logarithmic concentration scale, the time to $10\%$ conversion exponentially decays as a function of increasing initial Initiator concentration. This model fits our linear growth system data (Fig. 3A).

In an exponential growth system, the total mass of polymer product, *P*, grows as a function of initial Initiator concentration, $I_0$, and time, *t*, as follows:

3 $$\begin{aligned} P&= (I_0 + I_{leak}) e^{(kt)} . \end{aligned}$$

The time at which $10\%$ of monomers are consumed, $t_{10\%}$, is

4 $$\begin{aligned} t_{10\%}&= \frac{1}{k} (\ln (P_{10\%}) - \ln (I_0 + I_{leak})). \end{aligned}$$

Thus, in an exponential growth system, the time to $10\%$ completion of polymer growth ($10\%$ conversion of hairpins) is a linear function of the logarithm of the initial Initiator concentration. When plotted on a logarithmic concentration scale, the time to $10\%$ conversion linearly decreases with increasing initial Initiator concentration. This is what we observe in our exponential growth system data (Fig. 3B).

We quantify the leak via spectrofluorimetry experiments in Fig. 3^8^: we adjust the Initiator concentration [*I*] by an additional term $[I]_{leak}$ to obtain an effective Initiator concentration $[I]_{effective} = [I] + [I]_{leak}$. We then fit the $[I]_{leak}$ parameter to our data and find that in the exponential system $[I]_{leak} = 0.04\times$ and in the linear system $[I]_{leak} = 0.01\times$. Reactions were started with the addition of Hairpin 1 in order to avoid the leak. (The baseline in Fig. 3 contains all hairpins except for Hairpin1).

Equations 5 and 6 below correspond to Eqs. 2 and 4 but include the fit parameters.

5 $$\begin{aligned}{} & {} t_{10\%} = \frac{2.3(0.1 \cdot [H1]_0)}{[I]_0+0.01} \end{aligned}$$

6 $$\begin{aligned}{} & {} t_{10\%} = 0.3667 \ln \frac{0.1*[H1]_0}{[I]_0 + 0.04} + 1.245 \end{aligned}$$

The polymers formed at each Initiator concentration were examined by gel electrophoresis in order to characterize their length distribution. Each Initiator molecule was tagged with one ROX fluorophore. As the hairpins are successively added to a polymer, each polymer that is “properly initiated” retains exactly one fluorophore, thus the ROX fluorescence signal directly correlates to the number of polymers at a given size. The sizes were binned after post-staining with SYBR Gold, which allowed the DNA ladder to be visualized.

The mean length (in base pairs) of polymers decreases with increasing Initiator concentration above 4% of relative hairpin concentrations. (See Figs. S6, S7, S8 for gels and binned data of both linear and exponential systems). This is expected because high concentrations of Initiator outcompete existing insertion sites for free hairpins. In the case of Initiator concentration below 4% of relative Hairpin concentrations, the different amounts of leak in the systems are presumably responsible for the different distributions of polymer length between the linear and exponential system. The smaller leak in the linear system (1%) would explain why the linear system produces longer polymers than the exponential system (which has a 4% leak).

Atomic Force Microscopy of the reaction product confirms the formation of unbranched polymers in the exponential system (Fig. S9). In comparing images of both the polymer and the leak product, we find that the leak product is capable of growing much larger than the intended polymer, but the polymer grows faster. Others have shown that polymer growth in the absence of Initiator can provide an upper bound for how big the polymer can grow^34^. It is unclear whether the leak product is a linear polymer. It may be a highly pseudo-knotted structure.

### Time Lapse experiments

A qualitative difference between the exponential and linear systems is also observed when examining polymer size over time in Fig. 2. (See Figs. S10, S11 and S14 for three additional exponential system time lapse gels and see Figs. S12, S13 and S14 for the SYBR Gold stained versions of all of these gels). The bottom edge of the thick red lines in Fig. 2 indicate 1000 base pairs.

Several features of this gel data are worth noticing: First, the exponential system generates longer polymer products sooner than the linear system (Fig. 2), 500 min for the linear system and 60 min for the exponential system each to produce a 1000-base pair polymer. The exponential system produces a detectable amount of 1000-base pair polymer within 20 min, at least four times faster than the linear system, which takes between 90 and 120 min to produce a 1000-base pair polymer.

Second, the growth rate of the polymers for the exponential system increases drastically, starting at the 30-min point, as seen in the bottom right part of Fig. 2. The growth rate in the linear system is more steady, as seen in the top right part of Fig. 2).

Third, the length of the polymer tends to have a larger variation in the exponential system. Polymers of size over 2500 base pairs are generated while the average size is still far below 1000 base pairs. The distribution of polymer size is much closer to a normal distribution for the linear system. This phenomenon also fits the expectation for the exponential growth system: longer polymers can grow faster because they have more sites for the insertions to happen in parallel.

Although the above three features suggest but do not directly prove exponential growth, all of them are consistent with the expectation that exponential growth progresses. We note that the actual mean of polymer length is larger than that reported here, especially for the exponential system, because of our conservative binning of data (all gel smears above 3000 base pairs are lumped into a bin of size 3000 base pairs).

Figure 2B is particularly rich in data. In addition to showing that the polymers produced in the exponential system grow large quickly, the gel clearly shows that polymer growth occurs in quantized chunks of approximately 25 base pairs at a time. This is expected, as each hairpin contains between 54 and 57 nucleotides. The bands generated by the polymerization alternate between faint and dark within each lane. This corroborates our earlier claim that Hairpin 2 is slower to react with its insertion site than Hairpin 3. If the backward reaction rates for both of these reactions are equivalent, then this implies that the reaction between H2 and its insertion site is a slower step in the formation of polymers.

The exponential time lapse gel in Fig. 2 and the replicate in Fig. S11 expose an issue. The signal of the bands relative to background fades from left to right. In the SYBR Gold-stained versions of these gels, as shown in Fig. S13, the lanes to the right show noticeably less total stained DNA than the other lanes. We suspect that this behavior is a result of the complexity of loading the gel: in order to ensure that the experiments are initiated and the gel is run exactly on time, the right half of the gel (higher time point reactions) is loaded approximately 30 min in advance of the shorter time lapse reactions. This may allow for the DNA in these wells to diffuse out of the wells in advance of running. Another concern is the fading of the bands at the top of the gel in the longer time lapse reactions.

We hypothesize that the fluorescent loading dye bromophenol blue interferes with the fluorescence read-out of our properly initiated polymers. The gel in Fig. 2B has a dark band in all lanes across the bottom of the gel. By comparison, this band becomes faint at intermediate times for the replicate in Fig. S11 and disappears at long time points in the replicate in Fig. S10. In the SYBR Gold-stained versions of the gels in Fig. 2B and Fig. S11, as shown in Fig. S13, this band fades significantly. Since bromophenol blue does not fluoresce at the excitation spectra of SYBR Gold, we can assume that only stained DNA is visible, and that if the dark lower bands in the gels were unused initiator, then there would be a larger amount of DNA at these lengths. A more complete analysis of these gels was precluded due to the interference of the fluorescent loading dye bromophenol blue and an improperly stained ladder in the linear system time lapse gel that makes it difficult to resolve at molecular weights above 1000 base pairs (Fig. S12).

The chemical reactions at play in the exponential growth system are modeled and discussed in supplementary material Section S4.

## Methods to generate other behaviors

### Division

**Figure 4:**
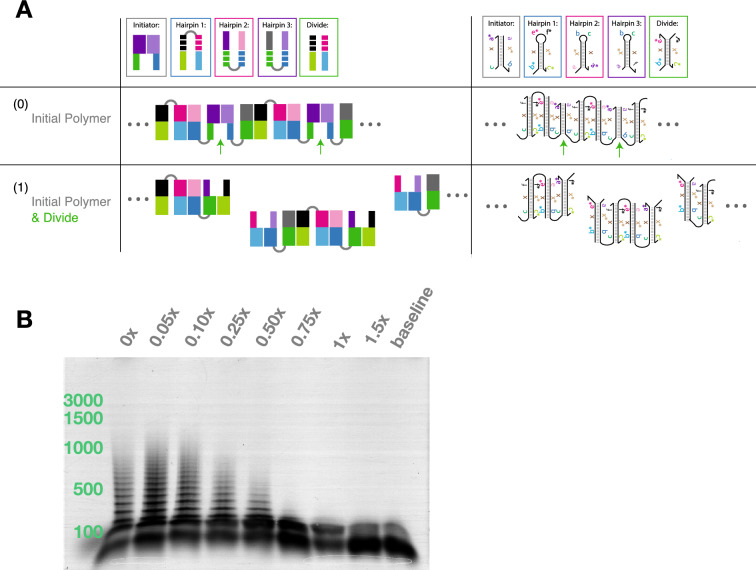
A system that implements division. (**A**) This figure depicts a system that implements division in a polymer. Each oligonucleotide is shown with color-coded motifs that correspond to the colored subsequences shown in Fig. 1.. The boxes around each oligonucleotide correspond to the insertion arrows in the mechanism below, which shows the insertion of two Divide complexes. The Divide complex is identical to Hairpin 1, except that the hairpin is split between domains e* and f*. (**B**) Super Fine Resolution Agarose non-denaturing gels of the product of a polymerization reaction with 80 nM ROX-labeled Initiator and 1 $\mu$M Hairpin 1, Hairpin 2, and Hairpin 3, to which Divide complex was added at concentrations $[D]_0 = \{ 0\%, 5\%, 10\%, 25\%, 50\%, 75\%, 100\%, 150\%\}$ relative to hairpin concentrations. Divide complex was added after 6 hours of reaction, and allowed to incubate for 100 min.The size of polymers decreases with increased concentrations of Divide complex. See Fig. S15(B) for gel after staining with SYBR Gold.

Just as a polymer can grow in logarithmic time via parallel insertion, a population of polymers can be generated in logarithmic time using insertional division. Division is implemented by a complex that is identical in sequence to Hairpin 1 except that its loop has a break in it (Fig. 1A). When this complex inserts itself into a chain, the polymer splits into two. Figure 4A illustrates the general scheme and its implementation in DNA sequences. Figure 4B shows that the division complex can can reduce the length of the polymers after they are formed.

### Treadmilling

When linear insertion is combined with end-point division, one behavior that emerges is “treadmilling”. Treadmilling is the condition in which there is growth at one end of a polymer while the other end is shrinking. Figure S23 shows a mechanism for treadmilling using the insertion system presented here. Note that we have not experimentally verified treadmilling. This design requires a different set of monomers and a different hairpin structure than what we have discussed and shown here. While our treadmilling mechanism utilizes both insertion and division, our design is incompatible with parallel insertion and division. A successful implementation of this mechanism would also require careful kinetic control over the insertion and division primitives.

We observe division of polymers when the Divide complex is added to the reactions six hours after initiation. We also observe short polymers when the Divide complexes are added to the solution at the beginning of the reaction, in which case they directly compete with exponential growth (Figs. S15, S16, S17, S18). We confirmed that monomer conversion is logarithmic in time at two different concentrations of Divide complexes (Figs. S19, S20, S21 and S22).

## Conclusions

This work is part of a growing push in nanotechnology and material science that is oriented toward fabricating smarter materials that can be programmed and created using molecular reactions. The molecular focus renders these structures as potentially capable of being interfaced with biological compounds and systems. Such materials are capable of complex behaviors and creating programmed and time-varying complex structures, but they will not run the same types of programs that computers run. Rather these molecular systems should be used to do what molecules do best: communicate and process in-vitro and in-vivo information via shape, structure, function and molecular interaction.

We have identified a new class of programmable behaviors that use a structured logic for moving molecules relative to each other, with the ability to modify the existing substructure. We constructed a computational model for *active self-assembly* that is sufficiently expressive as a Pushdown Automaton, to achieve these behaviors and is the first of its kind to be implemented in molecules. We designed and experimentally verified the construction of the first synthetic linear polymer capable of growing exponentially quickly and is also capable of dividing. It is an example of a powerful one-dimensional tool that allows engineers to change the interconnections of molecules after a shape has been assembled, which is an important step toward fully reprogrammable molecular assembly. We demonstrated two different types of behavior using a simple insertion primitive (insertion and splitting), and we have proposed a third behavior (treadmilling) that results from the composition of insertion and division but requires a slightly modified molecular implementation.

In the supplementary material section S1, we also observe that the exponentially-fast growth of molecular systems such as ours suggests a need for a new type of theoretical foundation—a physically-oriented extension of the traditional sequential “Computable Functions” found in theoretical Computer Science. This foundation would likely use “physics-of-computation” versions of abstractions that incorporate exponential parallelism, such as Boolean circuit theory and Parallel Random Access Machine (PRAM) theory^35–39^ that can gracefully incorporate the physical domain and physical goals and properties, as well as computational features.

As a result, we and other investigators have been seeing that there is a developing type of computational framework, which can be called “Extended Physical Computation”^40–42^. The new framework will include time, space, movement, rates of change, energy, yield, volume or mass, in addition to the the traditional computational abstractions, which act purely on abstract numerical functions over the integers. We feel that the existing formalisms do not capture enough of the essential ingredients that are needed, for instance, even for the study and foundations for exponential growth, in this combined physical and computational sense. The development of this new type of computational theory could help us understand theoretical issues of exponential growth of programmable physical structures, and would help resolve other theoretical issues for the fabrication and design of programmable self-assembled physical problems in chemistry, biology and robotics.

We feel that the connection of essential theory to “Extended Physical Computation” will become increasingly important to explore and develop, as molecular and self-fabricating technologies become progressively more applicable. As a result, we invite theorists to examine the foundations of these issues using physical world parameters, to develop interesting new types of fundamental results and new formalizations, to extend and combine the ideas of effective calculation and growth.

Our system is the first to implement “internal” parallel insertion and does not rely on adding layers to “external edges” for growth, and is also the first exponential structure that is linear and is not a tree. This feature of our system provides for improved exponential growth in our system, as our system is not as limited by the external surface area, although there will still be physical limits and constraints.

Our molecular system is not, however, the first exponentially fast growing structure ever synthesized. Yin et al. constructed a binary molecular tree out of DNA^8^. Their reaction begins with a root node, where each node generates two child nodes in each generation of growth. The authors point out that, in the absence of steric effects, a linear increase in the number of node species will yield an exponential increase in the size of the binary tree. In practice, steric effects are always present.

A next challenge will be to build reprogrammable molecular shapes in two and three dimensions. Difficulties are likely to arise when scaling our current molecular system to these dimensions. Until we can more precisely control the kinetics of hairpin insertion, we cannot guarantee the proper exponential growth of a shape in these higher dimensions. This is because our polymer is too flexible to accommodate insertions in multiple dimensions without the possibility of self-interactions forming a mis-shaped object. The generation of a well-formed object using an elaboration of our system will require component molecules with rigid structures.

A second limitation of our construction is the repeating DNA sequence utilized in the insertion and division primitives. In theory, these structures can be programmed just like tiles in the tile assembly model, but in practice the repeating DNA sequence places a constraint on how many different actions can take place at a given site. One may be able to extend this system by adding more complexity into the hairpin loops—additional structures or sequences that might accommodate other functionalities (akin to the way an amino acid’s functional group confers a variety of alternative interactions). The power of our system lies in its ability to grow a structure very quickly with only a few types of monomers by allowing subsets of molecules to move relative to each other. When a system like ours is scaled up its power would be limited, because Brownian motion drives these translocations only on small scales.

This work straddles the fields of synthetic biochemistry and theoretical computer science. The fields of synthetic biology and chemistry could greatly benefit from new methods to classify the complexity of molecular systems. There remain many unanswered questions at the intersection of these two sciences. Identifying and implementing the necessary primitives required to implement all active assembly behaviors that chemistry and biology have observed, will provide more insight into how nature works and how we can engineer it.

## Materials and methods

### Experimental system

A typical fluorescence kinetics experiment contains Hairpin 2 labeled with a fluorophore and quencher pair on the 3’ and 5’ ends of the strand, respectively. Mixed together with H2 are I, H1 and either the inactivated or regular version of H3 for the linear and exponential systems respectively. H1 is added last to trigger the reaction. As H2 is integrated into the polymer, the quencher and fluorophore pair are separated from each other, yielding an increased fluorescence signal in the solution. At the end of the experiment, another strand of DNA is added into the solution in order to fully displace all unreacted hairpins (Fig. S5). This “displacement” strand was added in $> 50 \times$ excess to the concentration of H2RQ to ensure that the reaction quickly goes to completion. We use the final fluorescence level to normalize our fluorescence signals. Baseline reactions contain only I, H2 and H3, until the end of the experiment at which point the displace strand is added.

### DNA sequences and design

The sequences presented in Tables B.1 are based on those used in a previous insertional polymerization motor^15^. These sequences were designed using the NUPACK web application^43,44^ and our in-house DNA Design software package^45^ to minimize the presence of any unanticipated secondary structures that might interfere with the kinetics under investigation.

### Buffer conditions

DNA oligonucleotides were stored in 1$\times$ SPSC buffer (50 mM Na2HPO4 pH 6.5, 1 M NaCl) at $4^{\circ }$C directly preceding experiments. All experiments and purifications were performed at $25^{\circ }$C.

### Annealing

All annealing processes were performed with an Eppendorf Mastercycler Gradient thermocycler. The samples were brought down from $95^{\circ }$C to $16^{\circ }$C at a constant rate over the course of 90 min.

### Snap cooling

All Hairpins were snap cooled prior to experiments. This protocol entails heating the strand solution to $90^{\circ }$C for 5 min, then immediately putting solutions on ice for 45 min. This protocol encourages intramolecular hydrogen bonding of the hairpins.

### Substrate purification

DNA oligonucleotides used in this study were purchased from Integrated DNA Technologies (IDT), with standard desalting purification, except for strands with a quencher, fluorophore or a $5^{\prime }$ toehold involved in the four-way branch migration, which were purchased with HPLC purification.

Concentrations of individual strand stocks were determined from the measured absorbance at 260 nM using a Nanodrop Biophotometer and using calculated extinction coefficients that account for hypochromicity effects in double-stranded DNA^46^.

Initiator and Divide complexes were further purified by nondenaturing (ND) polyacrylamide gel electrophoresis (PAGE) as follows: Strands for each sample were prepared with nominally correct stoichiometry at 10 nM and annealed. The acrylamide (19:1 acrylamide:bis) was diluted from $40\%$ acrylamide stock (Ambion). ND loading dye (containing Bromphenol Blue in $50\%$ glycerol) was added to all samples, achieving a final gycerol concentration of $10\%$ by volume. The samples were then run on $12\%$ ND PAGE at 150 V for 6 hours. Gels were run at room temperature ($\approx 25^{\circ }$C). The band corresponding to the Initiator size was cut out and eluted in 1 mL of $1\times$ SPSC buffer for 2 days. Purified complexes were quantitated by measurement of absorbance at 260 nm using an Eppendorf Biophotometer and calculated extinction coefficients as above.

### Gel assays

Combinatorial gels were run using 12% polyacrylamide and concentrations of all species at 100 nM. Solutions were left to react for 6 hours, then run in an XCell SureLock Mini-Cell Electrophoresis vertical gel box at 150V for 1 hour in TBE running buffer. After a gel was run, it was stained with SYBER Gold dye and imaged using an FLA-5100 fluorescent scanner (Fujifilm Life Science). Time Lapse, Final Value and Divide gels were run in 2% Super Fine Resolution Agarose (from AMRESCO) on a Thermo Scientific Owl Horizontal Gel box. In these experiments, the Initiator is tagged with a 3’ ROX fluorophore on one strand. Thus each properly-initiated polymer has a single ROX tag. Time Lapse reactions contained the following concentrations of species [I] = 80nM, [H1] = 1.5 $\mu$M, [H2] = 1 $\mu$M, [H3] = 1 $\mu$M. Final Values reactions contained the following concentrations of species [I] = 0 nM, 10 nM, 20 nM, 40 nM, 80 nM, 160 nM, 320 nM, 640 nM, 1$\mu$M; [H1] = 1.50 $\mu$M; [H2] = 1 $\mu$M; [H3] = 1 $\mu$M. Divide reactions contained the following concentrations of species [I] = 80nM; [D] = 0 nM, 50 nM, 100 nM, 205 nM, 500nM, 750nM, 1 $\mu$M, 1.5 $\mu$M; [H1] = 1 $\mu$M; [H2] = 1 $\mu$M; [H3] = 1 $\mu$M. The Gel was run 100 min after the addition of the divide complex to all samples.

### Atomic force microscopy

Atomic Force Microscopy images of polymer taken with 10% Initiator (10 nM) relative to Hairpin (100 nM). 50$\mu$L of 1$\times$ TAE 12.5 mM Mg$^{++}$ was deposited on mica (from Ted Pella), followed by 1 $\mu$ L of 5 mM Nickel Acetate and 2uL of 500 nM polymer sample after 5 hours of reaction. The sample was then imaged using a VEECO Nanoscope III with a vertical engage J-scanner.

### Spectrofluorimetry studies

Spectrofluorimetry studies were done using a SPEX Fluorolog-3 (Horiba) with external water bath and 1.6 mL synthetic quartz cells (Hellma 119-004F). The excitation was at 584 nm, while emission was at 604 nm. In all spectrofluorimetry experiments, the total reaction volume was 1.5 mL, the temperature was $25^{\circ }$C, and 2 nm band-pass slits were used for both excitation and emission monochrometers. Experiments were conducted with an integration time of 10 seconds at 60 second intervals. Prior to each experiment, all cuvettes were cleaned as follows: each cuvette was rinsed 15 times in Milli-Q water, 5 times in $70\%$ ethanol, another 15 times in Milli-Q water, and finally once more in $70\%$ ethanol and then Milli-Q water. For the slit size, concentrations, and integration times used, no measurable photobleaching was observed. Exponential and linear reactions contained the following concentrations of species [I] = 0 nM, 1 nM, 2 nM, 4 nM, 8nM, 16nM, 32 nM, 64 nM, 100 nM; [H1] = 150 nM; [H2] = 100 nM; [H3] = 100 nM. Divide reactions contained the following concentrations of species [I] = 10nM, 25 nM; [D] = 0 nM, 1 nM, 16 nM, 100 nM; [H1] = 150 nM; [H2] = 100 nM; [H3] = 100 nM.

### Fluorescence normalization

Fluorescence is normalized so that one normalized unit of fluorescence corresponds to 1 nM of unquenched fluorophore-labeled strand reporter 2. This normalization is based on the fluorescence levels of annealed samples with a minimal fluorescence measurement taken of the diluted Reporter complex before the experiment was initiated, and a maximal fluorescence value that is extracted from a biexponential fit of the data taken at the end of the experiment, after the displacement strand is added to displace all unreacted fluorophore-quencher pairs.

### Significance of results

In this paper, we demonstrate a molecular system for exponentially fast programmed growth using an “internal” insertion approach, which is less limited by physical interference than methods that passively rely on “external” accretion. Exponential growth aids future bottom-up fabrication—the self-manufacture of 2D and 3D molecular devices from simpler components. There is also a discussion in the supplementary material, which explains that an “extension” beyond conventional Turing machine theory is needed to analyze exponential growth in *programmable physical systems*, a task for theoreticians to explore. Physical sequential Turing machines cannot achieve exponential growth of structure in real time, yet taxonomically less powerful physical formalisms can do this. A need for an “extended” computation/physical theory arises.

## Supplementary Information

Supplementary Information.

## Data Availability

All data generated or analysed during this study are included in this published article and its supplementary information files.

## References

[1] Karsenti E (2008). Self-organization in cell biology: A brief history. Nat. Rev. Mol. Cell Biol..

[2] Yin P, Yan H, Daniell XG, Turberfield AJ, Reif JH (2004). A unidirectional DNA walker that moves autonomously along a track. Angew. Chem. Int. Ed..

[3] Tian Y, He Y, Chen Y, Yin P, Mao C (2005). A DNAzyme that walks processively and autonomously along a one-dimensional track. Angew. Chem. Int. Ed..

[4] Bath J, Green SJ, Turberfield AJ (2005). A free-running DNA motor powered by a nicking enzyme. Angew. Chem..

[5] Pei R, Taylor SK, Stefanovic D, Rudchenko S, Mitchell TE, Stojanovic MN (2006). Behavior of polycatalytic assemblies in a substrate-displaying matrix. J. Am. Chem. Soc..

[6] Green SJ, Bath J, Turberfield AJ (2008). Coordinated chemomechanical cycles: A mechanism for autonomous molecular motion. Phys. Rev. Lett..

[7] Omabegho T, Sha R, Seeman NC (2009). A bipedal DNA Brownian motor with coordinated legs. Science.

[8] Yin P, Choi HMT, Calvert CR, Pierce NA (2008). Programming biomolecular self-assembly pathways. Nature.

[9] Lund K, Manzo AJ, Dabby N, Michelotti N, Johnson-Buck A, Nangreave J, Taylor S, Pei R, Stojanovic MN, Walter NG (2010). Molecular Robots Guided by Prescriptive Landscapes. Nature.

[10] Muscat RA, Bath J, Turberfield AJ (2011). A programmable molecular robot. Nano Lett..

[11] Seelig G, Soloveichik D, Zhang DY, Winfree E (2006). Enzyme-free nucleic acid logic circuits. Science.

[12] Zhang DY, Turberfield AJ, Yurke B, Winfree E (2007). Engineering entropy-driven reactions and networks catalyzed by DNA. Science.

[13] Win MN, Smolke CD (2008). Higher-order cellular information processing with synthetic RNA devices. Science.

[14] Dirks RM, Pierce NA (2004). Triggered amplification by hybridization chain reaction. Proc. Natl. Acad. Sci..

[15] Venkataraman S, Dirks RM, Rothemund PWK, Winfree E, Pierce NA (2007). An autonomous polymerization motor powered by DNA hybridization. Nat. Nanotechnol..

[16] Dabby, N., & Chen, H.-L. Active self-assembly of simple units using an insertion primitive. In: *Proceedings of the Twenty-Fourth Annual ACM-SIAM Symposium on Discrete Algorithms*, 1526–1536 (SIAM, 2013).

[17] Hescott B, Malchik C, Winslow A (2015). Tight Bounds for Active Self-Assembly Using an Insertion Primitive. Algorithmica.

[18] Winfree, E. On the Computational Power of DNA Annealing and Ligation. In *DNA Based Computers* (eds Lipton, R. J. & Baum, E. B.) 199–221 (American Mathematical Society, Providence, RI, 1996).

[19] Feynman RP (1982). Simulating physics with computers. Int. J. Theor. Phys..

[20] Lloyd S (2000). Ultimate physical limits of computation. Nature.

[21] Rothemund PWK, Papadakis N, Winfree E (2004). Algorithmic self-assembly of DNA Sierpinski triangles. PLoS Biol..

[22] Barish RD, Schulman R, Rothemund PWK, Winfree E (2009). An information-bearing seed for nucleating algorithmic self-assembly. Proc. Natl. Acad. Sci..

[23] Woods Damien, Doty David, Myhrvold Cameron, Hui Joy, Zhou Felix, Yin Peng, Winfree Erik (2019). Diverse and robust molecular algorithms using reprogrammable DNA self-assembly. Nature.

[24] Demaine E, Demaine M, Fekete S, Ishaque M, Rafalin E, Schweller R, Souvaine D (2008). Staged self-assembly: Nanomanufacture of arbitrary shapes with o(1) glues. Natural Computing.

[25] Demaine E, Eisenstat S, Ishaque M, Winslow A (2012). One-dimensional staged self-assembly. Nat. Comput..

[26] Chen, H. L., & Doty, D. Parallelism and time in hierarchical self-assembly. In: *Proceedings of the Twenty-Third Annual ACM-SIAM Symposium on Discrete Algorithms*, 1163–1182 (SIAM, 2012).

[27] Cheng Q, Aggarwal G, Goldwasser M, Kao M-Y, Schweller R, Moisset de Espanes P (2005). Complexities for generalized models of self-assembly. SIAM J. Comput..

[28] Padilla, J., Patitz, M., Pena, R., Schweller, R., Seeman, N., Sheline, R., Summers, S., & Zhong, X. (2013). Asynchronous signal passing for tile self-assembly: Fuel efficient computation and efficient assembly of shapes. In *Proceedings of the International Conference on Unconventional Computation and Natural Computation*, 174–185.

[29] Woods, D., Chen, H.-L., Goodfriend, S., Dabby, N., Winfree, N., Yin, P. Active self-assembly of algorithmic shapes and patterns in polylogarithmic time. In *ITCS 2013: Innovations in Theoretical Computer Science*, 353–354, Berkeley, CA (ACM, 2013)

[30] Hescott, B., Malchik, C., & Winslow, A. Non-determinism reduces construction time in active self-assembly using an insertion primitive. In *Computing and Combinatorics: 24th International Conference, COCOON 2018, Qing Dao, China, July 2-4, 2018, Proceedings*, 626–637 (Springer, 2018).

[31] Yurke B, Turberfield AJ, Mills AP, Simmel FC, Neumann JL (2000). A DNA-fuelled molecular machine made of DNA. Nature.

[32] Zhang DY, Winfree E (2009). Control of DNA strand displacement kinetics using toehold exchange. J. Am. Chem. Soc..

[33] Dabby, N. *Synthetic molecular machines for active self-assembly: prototype algorithms, designs, and experimental study*. PhD thesis, California Institute of Technology (2013).

[34] Beck, V. Personal communication (2011).

[35] Cook Stephen A, Dymond Patrick W (1993). Parallel pointer machines. Comput. Complex..

[36] Immerman N (1989). Expressibility and parallel complexity. SIAM J. Comput..

[37] Knuth, D. E. *The Art of Computer Programming: Volume 3: Sorting and Searching* (Addison-Wesley, 1997).

[38] Blelloch GE (1996). Programming parallel algorithms. Commun. ACM.

[39] Hillis, W. D. & Steele Jr., G. L. Data parallel algorithms. *Commun. ACM* **29**(12), 1170–1183 (1986).

[40] Maclennan BJ (2003). Transcending turing computability. Mind. Mach..

[41] Schaeffer, L. A physically universal cellular automaton. In *Proceedings of the 2015 Conference on Innovations in Theoretical Computer Science*, 237–246 (2015).

[42] Cotogno P (2003). Hypercomputation and the physical church-turing thesis. Br. J. Philos. Sci..

[43] Zadeh JN, Steenberg CD, Bois JS, Wolfe BR, Pierce MB, Khan AR, Dirks RM, Pierce NA (2010). NUPACK: Analysis and design of nucleic acid systems. J. Comput. Chem..

[44] Zadeh JN, Wolfe BR, Pierce NA (2011). Nucleic acid sequence design via efficient ensemble defect optimization. J. Comput. Chem..

[45] Winfree, E. DNA design toolbox. http://www.dna.caltech.edu/dnadesign/ (2012).

[46] Tataurov AV, You Y, Owczarzy R (2008). Predicting ultraviolet spectrum of single stranded and double stranded deoxyribonucleic acids. Biophys. Chem..

