# Supplementary material for: Molecular system for an exponentially fast growing programmable synthetic polymer
Source: Sci Rep. 2023 Jul 12;13:11295. doi: 10.1038/s41598-023-35720-5 (PMC10338630; doi:10.1038/s41598-023-35720-5)
Supplement: Supplementary file 1 — Supplementary Information. [file 41598_2023_35720_MOESM1_ESM.pdf]

# Supplementary Material: Molecular System for an Exponentially Fast Growing Programmable Synthetic Polymer

Nadine Dabby[1], Alan Barr[1], and Ho-Lin Chen[*2]

[1]California Institute of Technology
[2]National Taiwan University

This Supplementary Material is divided into several parts. Section S1 is a discussion growing out of our paper. The analysis of the exponential growth molecular system starts from section S2. Details of our experiments start from section S4

## S1 Discussion: Extensions to Physical Computation

This section is a discussion growing out of our paper. We observe that an "extension" beyond conventional Turing machine theory is needed to theoretically analyze exponential growth in *programmable physical systems*, to be a task for theoreticians to explore. Sequential physical Turing Machines that run a roughly constant number of Turing steps per unit time cannot achieve exponentially fast growth of structure. In contrast, the "active" self-assembly model presented in our paper, computationally equivalent to a Push-Down Automaton, is exponentially fast when implemented in molecules, but is *taxonomically less powerful* than a Turing machine. In this way, a physical Push-Down Automaton can be *more powerful* than a sequential physical Turing Machine, even though the Turing Machine can compute any computable function. A need arises for an "extended" computational theory for programmable physical systems. It could aid in the design and construction of the complex programming found in synthetic developmental biology, in the growth and computation needed to go from single cells to whole new designed organisms and molecular systems.

As we discussed in the paper, molecular programming, nanotechnology and synthetic biology raise the prospect of bottom-up fabrication, the manufacture of complex devices that assemble themselves from simpler components. Natural biological systems fabricate structures with enormous scale and complex behaviors, defined at atomic-scale resolution, which can grow quickly with small programs relative to their object size and algorithmic complexity [13]. A goal in the molecular synthesis field is to build biophysical systems with great complexity and power, with applications to medicine, the environment, and green manufacturing.

In our paper, we demonstrated the first molecular system for exponentially fast growth using a programmable "internal" insertion approach, which is less limited by physical interference than methods that passively rely on "external" accretion. Our

*Corresponding author.

molecular insertion methods provide an early step toward efficiently building complex two and three dimensional physical shapes in logarithmic time.

Some readers may be used to seeing exponentially fast growth in branched polymers [23], as a reviewer pointed out to us. In our Part 1 work, we created programmed exponentially fast growth in a linear molecular chain, where exponential growth is possible because the number of the programmatically-created internal insertion points in the chain is also growing, exponentially quickly over time.

In this section, we continue, and note a perhaps obvious but significant difference in capability between *programmable physical* systems and *purely abstract, unphysical*, purely theoretical computational systems.

We note that a sequential Turing machine when implemented physically, simply cannot achieve exponentially fast growth of structure in real time, even if it is programmed to implement something that increases exponentially. When implemented as a physical, sequential system, the algorithm will slow down and implement itself progressively more slowly, slower and slower. On a physical sequential Turing machine, exponentially fast growth over physical time is not possible.

We demonstrate an example of this issue with a simple recursive algorithm doubling memory use, in Figure S2, with plots of its performance on a laptop computer, of time, memory and iteration count.

The scientific value of Figure S6, Figure S7 and Figure S8 is to contrast **physical time** which is linear in operating time on the physical laptop versus **iteration count**, which would not need any time at all to run, on an abstract unphysical machine. We would use the counter as a proxy for time. The algorithm is exponential in terms of the growth of structure. The differences between these simple graphs can be a surprisingly subtle issue, even for such a simple algorithm.

Thus, there can be a "tension" between the "physical" vs the unphysical (or purely abstract or theoretical) computational systems. This difference, which can be obvious in hindsight for some applications, requires a type of "extension" to the traditional abstract computational theories, for analyzing exponentially fast physically implemented computational systems.

For instance, natural biological systems and many other natural physical systems can grow exponentially fast over time. As a result, they cannot be implemented as physical, sequential Turing machines. They would need to be implemented in some other way, and would require a new type of robust theory of their own, for robust analysis.

As a result, this section tries to carefully motivate the need for an "extension" beyond abstract Turing machine theory, to extend the fundamental model of computation in this context. The extension would add the real-world's physical properties like physical time, energy, and other physical properties into the fundamental theory, as first class members of the new theory for programmable *physical* systems, as distinct from programmable *abstract* systems.

We claim that there is a need to theoretically analyze exponentially fast growth in programmable physical systems to create this new type of theory, as a future task for theoreticians to explore. It would create a new body of work that would build on the current abstract model of computation and extend it for programmable physical systems, and could aid in understanding and controlling exponentially fast growth of programmable physical systems. This new type of theory would aid our understanding and could have the potential to improve reaction yield and performance for many practical applications of bottom-up self-fabrication.

**Exponential growth in Natural Biological Systems.** In natural biological systems, periods of programmed exponential growth per unit time are com-

mon, and is perhaps almost ubiquitous. Understanding and controlling exponential growth will become key, to obtain acceptable reaction yield and performance for practical applications of bottom-up self-fabrication.

The task in this section grew out of what was a surprising observation for us at first. We saw that the molecular insertion system we designed, computationally equivalent to a Pushdown Automaton, when implemented in molecules as described in the paper perform exponential growth tasks that most Turing-Complete molecular systems could *not* perform, such as the DNA Tile Assembly Model [20], even though Turing Complete systems can in principle, implement any computable function. The Turing-Complete DNA Tile system cannot achieve "exponentially quick" growth in real physical time, while the Pushdown Automaton in Figure 1C can, even when both are implemented molecularly. Yet the Turing-Complete molecular system can, in theory, compute "anything." In our molecular system, the entire growth of a linear polymer resembles generating a string using a context-free grammar, with the rules executed in parallel and exponentially fast. The growth process of a polymer has the same expressive power as a single execution of a pushdown automata but executes exponentially faster, and thus cannot be simulated by a sequential Turing machine. Although the insertions taking place at different insertion sites do not directly affect each other, they produce the final output (the linear polymer) and its pattern collectively. Also, each insertion operation may determine whether more insertion sites can be produced or not, and can push other insertion sites farther apart. This is analogous to generating strings in a context-free grammar in which the application of grammar rules to different elements do not affect each other, but different applications of the grammar rules are still considered as the same 'computation' which produces the final output string. Previous work [7, 10] has also shown that the insertion primitives are equivalent to grammar rule applications in a context-free grammar.

**A Need for a new computational/physical abstraction.** The previous observation led us to see a need for a new type of combined physical/computational theory, to be called "Extended Physical Computation," intended to be suitable for understanding *programmable physical systems*, especially in the context of exponential growth.

This relates to the the body of work initiated in the 1980's by Carver Mead, John Hopfield and Richard Feynman, on the Physics of Computation [9, 15]. This group noted that all "actual" computation is physical, of course, even though there was a purely mathematical abstraction as a foundation.

But by focusing on the concept of the "extension" in this paper, however, we hope to explain why developing this new body of theoretical work would be useful. It can become one of the keys for understanding and controlling exponential growth and for obtaining more favorable reaction yield and performance for practical applications of bottom-up self-fabrication.

A new type of theoretical and conceptual framework could be useful for understanding how to build and analyze exponentially-fast, complex, programmable physical systems, analogous to the periods of exponential growth found in many natural biological systems.

**Extensions to Physical Computation.** We use the term "Extended Physical Computation" or "Extensions" because the term "Physical Computation" had already been used in the literature to denote "physical realizations of purely abstract computations" [17, 8], and the "Physics of Computation" term from the 1980's was perhaps too similar. An "extension" is needed because traditional Turing computing theory includes functions and integers, but doesn't include physical time and other physical

quantities. Exponential growth in real time doesn't fit within the Turing computing theory. (Turing himself initiated the studies of chemical morphogenesis in 1952 [19, 18] and perhaps could have extended computability theory, had he survived longer.)

Many exponential algorithms run exponentially slowly when implemented on a digital computer (for example, see the recursive data-structure algorithm shown in Figure S2 and the results in Figures S3 and S4). A new type of combined physical/computational theory would include traditional computational elements, like functions, inputs and outputs and binary numbers, similar to the traditional Turing theory, but would also include *physical quantities* as "first-class citizens," such as real-world kinetic and potential energy, physical time, physical space, geometric movement, entropy, momentum and angular momentum, emission of light and other physical items like mass, chemical yield, and other physical real-world quantities.

## S1.1 Discussion for "Extended Physical Computation"

In the fields of amorphous computing and reservoir computing, the term "Physical Computation," is often used to refer to "the physical realization of an abstract computation" and its properties [1, 17, 8, 16]. In that body of work, the authors compare the computational power of different *physical computation* systems in which the traditional computable functions and integers are used as the underlying objects for their studies. Their intent is to try to measure and compare the complexity and resource requirements of these physical computation systems, to simulate the computational power of different abstract and combinatorial computations, as implemented physically.

In contrast, in section we propose benefits coming from an augmentation of this term, an "Extended" type of Physical Computation, where additional basic objects and primitives are to be added as the fundamental objects of study, in addition to the computable functions and the integers. In this new type of "extended" physical computational theory, new computation-like primitives are added, which are found in the physical world, *physical quantities* such as kinetic and potential energy, physical time, physical space, movement, entropy, physical emission of light, and other physical things like mass, chemical yield, and other physical real-world quantities.

**An Unexpected Starting Point.** A starting point for the new computational theory was a surprising observation for us. We saw that the molecular insertion system we designed is computationally equivalent to a Pushdown Automaton, implemented in molecules as described in Figure 1C could perform tasks that certain Turing-Complete molecular systems could not perform, such as the DNA Tile Assembly Model [20]. The Turing-Complete DNA Tile system cannot achieve "exponentially quick" growth in real physical time, but the Pushdown Automaton in Figure 1C can, even when both are implemented molecularly. Yet the Turing-Complete molecular system can, in theory, compute "anything," i.e., any computable function. This was surprising to us at first, since a Turing-Complete system is more powerful, in a purely computational sense, than a Push-Down Automaton, which is *not* Turing complete.

We then noted that any sequential physical Turing Machine that runs a roughly constant number of Turing steps per unit time cannot achieve exponential growth of structure per unit time. It would need to perform an exponentially larger and larger number of operations per Turing cycle of the machine, to achieve an exponential growth of structure, say memory, per unit time. We show a simple recursive algorithm exhibiting issues like this, shown in a recursive algorithm described in Figure S2.

With further thought, however, we noted that there

are more recent models of computation that can incorporate exponential parallelism, such as Boolean circuit theory and Parallel Random Access Machine (PRAM) theory [4, 12, 14, 3, 11]. We feel that these computational theories can likely be extended to work better with the physical domain, including molecular assembly and computation. That type of physically inclusive theory has not yet been developed extensively, but we feel that there can be an increasing benefit in doing this type of new work.

Even so, some of the Turing complete molecular examples can *not* achieve exponential growth per unit time, while other of the Turing *incomplete* molecular examples *c*an, even when both are implemented molecularly. Something beyond the traditional theory is needed to address this issue. We highlight molecular assembly and computation examples in Figure S1, where we sketch out part of an Extended Physical Computation taxonomy. The purpose of the figure is to help motivate the need for studying a new, Extended Physical Computation category of computation, since the exponential growth of structure for programmable matter does not fit well enough into the traditional computational theories.

There are several behaviors that are not "computations" in a classical sense. Examples include exponential growth and molecular motion relative to a surface. In the DNA tile system, the tiles cannot implement these behaviors because (a) there is no instruction for moving or rotating a tile relative to a surface and (b) passive self-assembly is exponentially slower than active self assembly. The nubot model [22], is a theoretical proposal that if molecularly, implemented, could achieve exponential growth.

**A Partial Taxonomy.** In the following figure, the *oval* $\mathbb{P}$ on the left represents "Extended Physical Computation," for programmable *physical* systems, and uses an abstraction that includes issues of energy, mass, physical time (*not* merely simulation numbers standing for time on the computer or an iteration count or integer on the computer), physical movement, physical emission of light, chemical "yield" and other physical issues and quantities.

Toward the right, an overlapping oval, $\mathbb{T}$, represents the "Traditional" or "Turing" classes of Computable Functions. Some molecular algorithms are sequential Turing compatible, and these behaviors are subsets of $\mathbb{T}$, and could not achieve exponential growth of structure per unit time when implemented physically.

The enclosing rectangular box represents an abstract universal set $\mathbb{U}$ for *all types* of computing-related theories, new or old.

The blue subset of oval $\mathbb{P}$ is for physical computation systems that include physical elements that are incompatible with traditional Turing computability theory and fall outside of oval $\mathbb{T}$.

The white subset that falls within the intersection of both ovals includes such things as electronic computers and related macroscopic electronic devices, which are physical devices. But their behavior also falls into the white subset of extended physical computation oval $\mathbb{P}$ that is also within the traditional Turing oval, $\mathbb{T}$.

A series of molecular systems are indicated in the figure as dots where we put molecular walkers outside the Turing region, due to the need for physical movement. Their *physical* behavior isn't found in computability theory; they fall within the blue subregion of $\mathbb{P}$. Exponentially fast growing systems also fall *outside* of oval $\mathbb{T}$.

We feel that new theoretical studies of Extended Physical Computation would be useful, and could help shed light on which algorithms can and cannot achieve exponential growth in molecular and other programmable physical systems.

To contrast physical time versus simulation time, or to contrast physical time with an iteration count

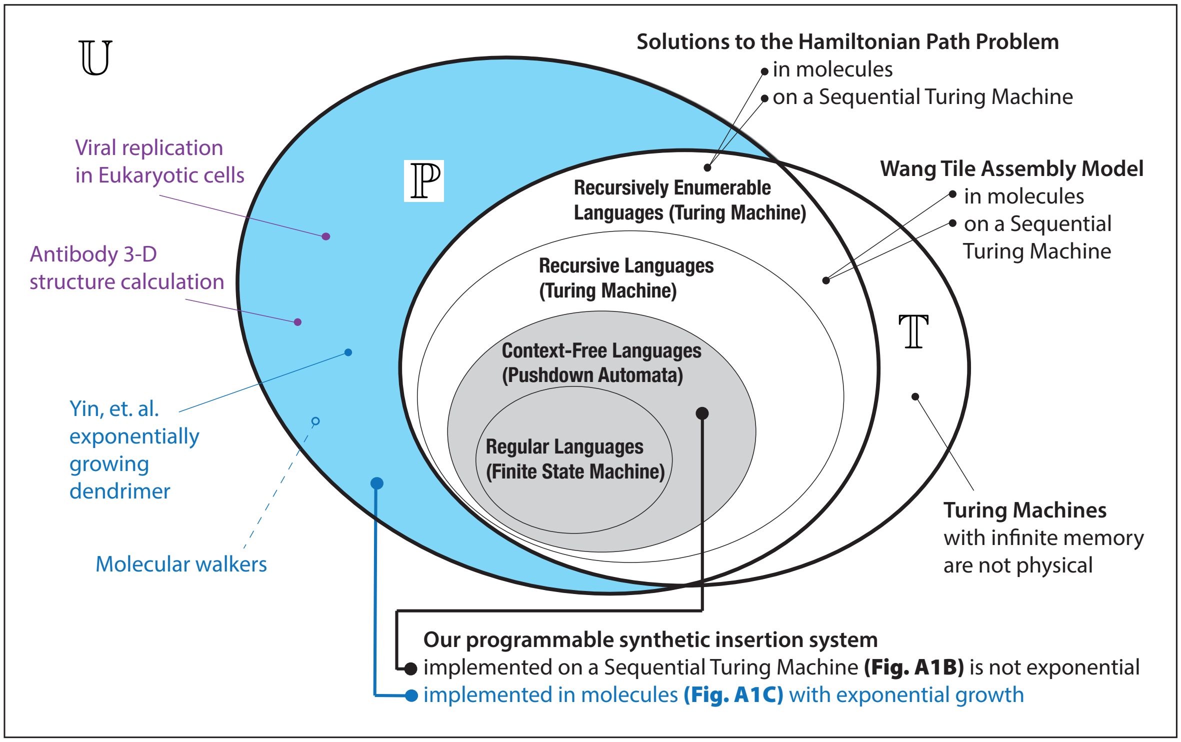


Figure S1: **A "Dividing Boundary," for Physical, Molecular Algorithms.** A key observation in the figure is that physically implemented algorithms that exhibit exponential growth per unit time *must* lie strictly outside the oval for the sequential Turing computing region $\mathbb{T}$. A full oval $\mathbb{P}$ stands for Extended Physical Computation. Exponentially quick and "necessarily physical" algorithms fall into the blue subset outside of $\mathbb{T}$. Some molecular algorithms, when implemented in molecules *can* achieve exponential growth of structure per unit real time, but can *not* grow exponentially quickly, when simulated physically on sequential computers (due to the fixed number of Turing steps per second). We have highlighted this issue in blue and black dots in the above figure, for our molecular algorithm described in Figure 1B and 1C. Other molecular algorithms, however, such as the DNA Wang tile model or molecular solutions to the Hamiltonian path problem, are sequential Turing compatible, and can *not* achieve exponential growth of structure per unit time, whether they are implemented in molecules, or else simulated on a sequential computer, highlighted with black dots. We classify a few other algorithms as dots within the figure.

on a digital computer, we wrote a simple computer program that doubles the amount of memory it uses, with each iteration in a loop. We plotted the amount of memory the program uses versus the iteration number, and also plotted the amount of memory the program uses versus physical wall-clock time. The memory use is exponential with the iteration number, since the memory doubles with each iteration in the loop. However, the memory use with physical time is *not* exponential, and is basically linear, using N operations per second. In the next section we show the computational memory-doubling example, shown in Figure S2 and plots of its performance.

**A New Abstraction is needed.** The observa-

tion about the differences in capability between the molecular/physical implementations and a pure computational abstraction, suggests a need for a new type of abstraction and a new type of computational theory that incorporates physical properties. This new abstraction could grow out of physics of computation-related work that can incorporate exponential parallelism, such as Boolean circuit theory and Parallel Random Access Machine (PRAM) theory, but would be extended so as to accommodate the physical domain. As a key difference, as we've said, a Traditional Sequential Turing-Complete system can compute any computable function, but most Turing-Complete molecular systems are *not* capable of exponential growth in real time, while other non-Turing-complete molecular systems like ours *can* exhibit exponential growth in real time.

As shown below in Figure S1, there have been two main types of research in the molecular structure and computation field, for instance, (a), where some researchers are translating traditional Computer Science problems into a molecular form, such as molecular Turing complete computation [21], and Hamiltonian path implementation [2], and also (b) where other researchers are creating novel molecular structures and behaviors, but without connecting the work as closely to traditional theoretical problems in Computer Science.

We feel that there is an increasing need to unify this "extended" body of theoretical work, to identify and study new, key properties within a new type of theory. We invite theorists to examine the foundations of these issues using physical world parameters, to try to develop interesting new types of fundamental results and new formalizations.

## S1.2 Example Algorithm

In this section, we provide a simple example in the context of exponential growth of structure per physical unit time. We will observe that exponential growth of structure per unit time does not occur on a traditional (Turing) electronic computer, even though exponential growth of structure takes place per "iterator" in a computational loop, as an iterator "$i$" goes from 1 to $N$. The exponential growth of structure per unit time cannot take place within any physical system that is equivalent to a sequential Turing Machine, even if implemented molecularly, where there is a roughly constant number of Turing steps per unit time.

In this example we construct a very simple recursive function that exponentially increases the size (in memory) of a data structure on a physical electronic computer with each successive iteration.

The input is an integer and the output is an exponentially increasing list of lists of 1's, in computer memory.

As a start, we seed the $0^{th}$ iteration as a short one-element list containing just the number 1. For the subsequent values, we recursively make a two-element list that consists of (two) **copies** of the value of the previous, $n-1^{th}$ iteration, in memory.

Here is the algorithm. The base case starts at $n = 0$. A recursive step for larger integers puts two **copies** of the previous generation into a new list.

**makelist(0)** $=$ **{1}**
**makelist(n)** $:=$ **{makelist(n-1), makelist(n-1)},**
$\mathbf{n \geq 1}$

Figure S2: **Algorithm 1.** Recursive algorithm to make exponentially growing copies of list data in memory, exponentially growing with an iteration integer, $n$, $n \geq 0$.

The total number of elements needed will double with each iteration. Each iteration will be a list, or a list of lists, or a list of lists of lists, etc., where the total memory in the data structure roughly doubles on each step.

In constructing this algorithm, the goal is to make

exponentially larger copies of data structures in memory as an analog or placeholder for making exponentially large growing systems of molecules and similar things found in algorithmic assembly and physical computation; the $1's$ can stand for individual molecules.

If this algorithm were implemented with something like pointers instead of copies, however, then we would scarcely need any new memory to be allocated at all! For the example to be more relevant, we need to use copies and shouldn't use pointers, to create exponentially increasing-size structures in memory.

Likewise, as an example algorithm, we could have chosen to use an exponential representation of a number system on a computer, to print out the numbers 1, 10, 100, 1000, which exponentially increase by a factor of 10. This choice also would not be a suitable analog for creating exponentially growing molecules and other growing physical structures. This digital algorithm represents an exponentially growing "thing" (a larger and larger number), but the representation itself is not exponentially growing, adding exactly one additional symbol, the digit "0."

We could even have the computer print symbols for an increasing series of transfinite cardinal numbers, $\aleph_0$, $\aleph_1$, $\aleph_2$, for quantities that grow infinitely quickly. This choice is also not a suitable molecular growth analog, since it prints out only two symbols per iteration.

Instead, our algorithm attempts to more faithfully mimic some of the essential aspects found in the exponential growth of molecular structure, in computer memory.

Figure S3 is some of the sample output from the recursive algorithm described in Figure S2.

$$i = 0: \quad \begin{pmatrix} 1 \end{pmatrix} \qquad i = 1: \quad \begin{pmatrix} 1 \\ 1 \end{pmatrix}$$

Figure S3: **Initial Output.** The output from the zero-th and first generation from the algorithm, for an iterator $i = 0$, and $i = 1$. When $i = 0$, the base case, the output is just the list $\{1\}$. The first generation consists of two copies of the zero-th generation. The reader can see the two 1's in the first generation, copied from the $0^{th}$ generation.

In figure S4, the reader can see that two copies of the "$i = 1$" case are in the "$i = 2$" case, and two copies of the "$i = 2$" case fall into the "$i = 3$" case.

The data structure size roughly doubles on each successive iteration, so it gets exponentially larger, with each iteration, doubling in size, for larger values of $i$.

$$i = 2: \left\{ \begin{pmatrix} 1 \\ 1 \end{pmatrix}, \begin{pmatrix} 1 \\ 1 \end{pmatrix} \right\}$$

$$i = 3: \left\{ \begin{pmatrix} 1 & 1 \\ 1 & 1 \end{pmatrix}, \begin{pmatrix} 1 & 1 \\ 1 & 1 \end{pmatrix} \right\}$$

Figure S4: **Next Cases.** Output from the second and third generation, for $i = 2$, and $i = 3$. Two recursive copies of the lists and the lists of lists are copied into each successive generation, roughly doubling its size.

| i | MEMSIZE | TIME |
|---|---|---|
| 1 | 88 | 0.00001 |
| 2 | 136 | $8. \times 10^{-6}$ |
| 3 | 232 | 0.000013 |
| 4 | 432 | 0.000023 |
| 5 | 832 | 0.000044 |
| 6 | 1600 | 0.000088 |
| 7 | 3208 | 0.000178 |
| 8 | $6.41 \times 10^3$ | 0.000353 |
| 9 | $1.28 \times 10^4$ | 0.000711 |
| 10 | $2.56 \times 10^4$ | 0.00145 |
| 11 | $5.12 \times 10^4$ | 0.00286 |
| 12 | $1.02 \times 10^5$ | 0.00572 |
| 13 | $1.97 \times 10^5$ | 0.0114 |
| 14 | $3.93 \times 10^5$ | 0.0233 |
| 15 | $7.87 \times 10^5$ | 0.0468 |
| 16 | $1.57 \times 10^6$ | 0.0964 |
| 17 | $3.15 \times 10^6$ | 0.192 |
| 18 | $6.29 \times 10^6$ | 0.386 |
| 19 | $1.26 \times 10^7$ | 0.815 |
| 20 | $2.52 \times 10^7$ | 1.6 |

Figure S5: **Data.** Data values from the algorithm, for iterator values "$i$" from 1 to 20. The memory grows exponentially with each iteration, seen in the middle column, memory vs iterator integer. Physical time, measured in seconds, is roughly linear with memory.

Figure S5 shows the amount of measured physical computer time (in seconds), per iteration, used to run this algorithm. The table and graphs show the amount of memory, and the time needed, in seconds, to construct the first 20 generations of this recursive data structure on a laptop computer.

The memory grows exponentially with each iteration, seen in the middle column, memory vs iterator integer. Physical time is roughly linear with memory.

It is clear from figure S6 that the data structure in the algorithm does *not* grow exponentially fast on a computer, and not on any molecular system equivalent to a sequential Turing machine, where it is assumed that there is a roughly constant average amount of physical time per Turing step.

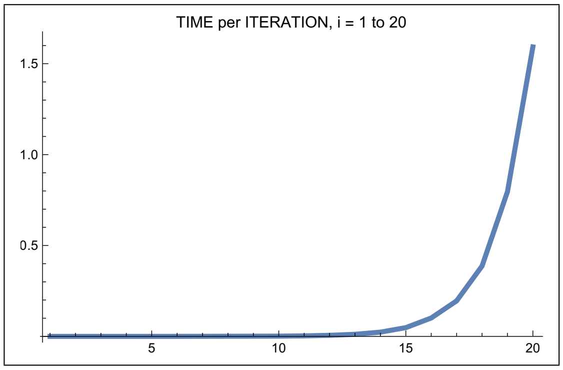


Figure S6: **Time in seconds** for running the recursive algorithm. It is not surprising that this algorithm takes longer and longer to run to create an exponentially larger data structure, for each successive run through the recursive loop, from $i = 1$, to $i = 20$. This is not an exponentially fast algorithm on a digital computer. In fact, each iteration is exponentially slower than the previous one.

As described in figures S7 and S8, the memory use is roughly exponential, in terms of growth per iteration. With regard to physical time, however, on a digital computer, the growth of this example structure is *not* exponential, and is only linear with time. This is in contrast to our exponentially fast molecular insertion algorithm shown in Figure 1 and other exponentially fast molecular algorithms shown in Figure S1, with exponentially fast growth of molecular structure per unit physical time.

The inability to create the exponentially fast growth of structure per unit time is to be expected on *any* physical system that is equivalent to a sequential Turing machine with a constant average speed per step. This example data structure algorithm will get **slower and slower**, in terms of when the next integer "$i$" will get executed, on the electronic computer, for each next iteration.

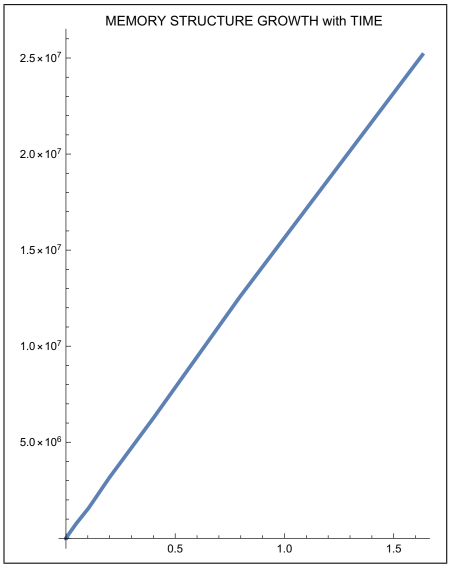


Figure S7: **Linear with time.** The memory growth of the recursive algorithm is roughly *linear* with physical time. In contrast, the molecular algorithm shown in Figure 1 from the companion paper is *exponentially fast* with physical time, when implemented molecularly, but *not* when implemented on a digital computer.

Similarly, the DNA tile system is *unable* to create an exponentially fast growth of structure per unit time, since it is equivalent to a sequential Turing machine. A roughly constant amount of physical time is available per Turing step. On a Turing Machine, an exponentially increasing number of steps would be needed, per unit time, for exponentially fast growth.

This inability of a Turing-equivalent system to perform exponentially quickly, where seemingly "lesser" systems can outperform them, like our insertion model, is one of the reasons why we felt that a new type of "Extended" Physical Computation Theory is needed.

A new body of theoretical knowledge of this type will help us better understand the fundamental abilities and limitations of self-assembling physical systems, for exponential growth and other important qualities. Some of the most relevant types of future programming may be found in synthetic developmental biology, for instance, in the growth and computation needed to go from a single cell to a new type of designed, whole organism.

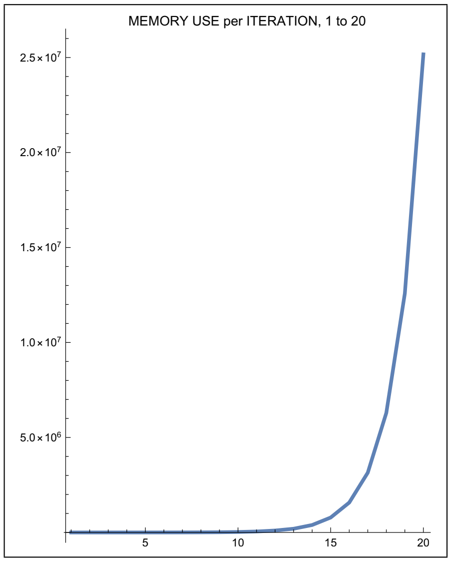


Figure S8: **Exponentially larger memory with iterator.** The memory growth of the recursive algorithm is exponential, however, with the *iterator index* from $i = 0$ to $i = 20$, roughly doubling on each iteration.

## S1.3 Conclusions

In this section, we observe that the exponentially-fast growth of molecular systems such as ours suggests a need for a new type of theoretical foundation – a physically-oriented extension of the traditional sequential "Computable Functions" found in theoretical Computer Science. This foundation would likely use "physics-of-computation" versions of abstractions that incorporate exponential parallelism, such as Boolean circuit theory and Parallel Random Access Machine (PRAM) theory [4, 12, 14, 3, 11] that can gracefully incorporate the physical domain and physical goals and properties, as well as computational features.

The new framework will include time, space, movement, rates of change, energy, yield, volume or mass, in addition to the the traditional computational abstractions, which act purely on abstract numerical functions over the integers. We feel that the existing formalisms do not capture enough of the essential ingredients that are needed, for instance, even for the study and foundations for exponential growth, in this combined physical and computational sense. The development of this new type of computational theory could help us understand theoretical issues of exponential growth of programmable physical structures, and would help resolve other theoretical issues for the fabrication and design of programmable self-assembled physical problems in chemistry, biology and robotics.

We feel that the connection of essential theory to "Extended Physical Computation" will become increasingly important to explore and develop, as molecular and self-fabricating technologies become progressively more applicable. As a result, we invite theorists to examine the foundations of these issues using physical world parameters, to develop interesting new types of fundamental results and new formalizations, to extend and combine the ideas of effective calculation and physical growth.

## S2 Molecular Analysis

The exponential growth molecular system described in this paper can be modeled with the following chemical reactions:

$$I1 + H1 \underset{k_{-1}}{\overset{k_1}{\rightleftharpoons}} S2 + S3, \tag{1}$$

$$S2 + H2 \underset{k_{-2}}{\overset{k_2}{\rightleftharpoons}} I2 + P2, \tag{2}$$

$$S3 + H3 \underset{k_{-3}}{\overset{k_3}{\rightleftharpoons}} I3 + P3 \tag{3}$$

where S2 and S3 are the insertion sites for H2 and H3 respectively, and P2 and P3 are double-stranded sections of polymer that are henceforth unreactive. The chemical reactions can be further simplified by the following three assumptions:

1. Each Initiation site is equivalent.
2. The forward rates are the same for all three reactions. Thus, $k_1 = k_2 = k_3 = k$.
3. The reactions are irreversible. Thus, $k_{-1} = k_{-2} = k_{-3} = 0$.

The first assumption makes the set of reactions tractable. The second assumption comes from the next section, where we show that the forward rate of a four-way branch migration reaction is dependent on the length and sequences of interacting toeholds and consequently on the overall free energy changes in the system. In our implementation all toehold pairs share the property of being nine bases long with approximately equivalent GC content, making their free energy changes roughly equivalent. The final assumption is justified by the decreasing free energy of the system at each step. We note that this assumption may not hold as reactants are consumed by the system.

With the above assumptions the set of reactions can be reduced to:

$$I + H1 \xrightarrow{k} S2 + S3, \tag{4}$$

$$S2 + H2 \xrightarrow{k} I + P2, \tag{5}$$

$$S3 + H3 \xrightarrow{k} I + P3. \tag{6}$$

We next require that [S2] = [S3], henceforth they will both be replaced by a variable called [S].

Then we can simulate the rate of change of [I], [H1], [H2], [H3], and [S] with the following differential equations:

$$\frac{d[I]}{dt} = -k[I][H1] + k[S][H2] + k[S][H3], \quad (7)$$

$$\frac{d[H1]}{dt} = -k[I][H1], \quad (8)$$

$$\frac{d[S]}{dt} = 2k[H1][I] - k[S][H2] - k[S][H3], \quad (9)$$

$$\frac{d[H2]}{dt} = -k[S][H2], \quad (10)$$

$$\frac{d[H3]}{dt} = -k[S][H3]. \quad (11)$$

The terms in the above equations (7, 9, 10 and 11) can be added together to establish

$$0 = 2 \times \frac{d[I]}{dt} + \frac{d[S]}{dt} + \frac{d[H2]}{dt} + \frac{d[H3]}{dt}, \quad (12)$$

or, equivalently,

$$\frac{d[S]}{dt} = -2 \times \frac{d[I]}{dt} - \frac{d[H2]}{dt} - \frac{d[H3]}{dt}. \quad (13)$$

Taking the integral of Equation 13 results in the solution $[S] = -2[I] - [H2] - [H3] + C$. Since the sum of [S], [I], [H2], and [H3] is a constant, we get:

$$[S]+2[I]+[H2]+[H3] = C = 2[I]_0+[H2]_0+[H3]_0, \quad (14)$$

$$[S] = 2[I]_0 + [H2]_0 + [H3]_0 - 2[I] - [H2] - [H3]. \quad (15)$$

Assuming that [H2] = [H3], and $[H2]_0 = [H3]_0$, this results in:

$$[S] = 2 \times ([I]_0 - [I] + [H2]_0 - [H2]), \quad (16)$$

and

$$[S] = 2 \times ([I]_0 - [I] + [H3]_0 - [H3]). \quad (17)$$

Finally, we use Equations 16 and 17 to simplify the set of differential equations (7 to 11) to:

$$\frac{d[I]}{dt} = -k[I][H1] + k[S][H2] + k[S][H3], \quad (18)$$

$$\frac{d[H1]}{dt} = -k[I][H1], \quad (19)$$

$$\frac{d[S]}{dt} = 2k[H1][I] - k[S][H2] - k[S][H3], \quad (20)$$

$$\frac{d[H2]}{dt} = -2k[H2]([I]_0 - [I] + [H2]_0 - [H2]), \quad (21)$$

$$\frac{d[H3]}{dt} = -2k[H3]([I]_0 - [I] + [H3]_0 - [H3]). \quad (22)$$

When simulated in Matlab, these equations result in a plot of the concentrations of I, H1, H2, and H3 over time. Figure S9 shows the simulation. The simulation starts with initial concentrations: $[I]_0 = 8$ nM, $[H1]_0 = 150$ nM, $[H2]_0 = [H3]_0 = 100$ nM as in our spectrofluorimetry experiments. We set $k = 5050M^{-1}sec^{-1}$, as derived from the spectrofluorimetry data shown in Figure 3 and described in the next section.

The simulated concentration of H2 over time shows $[H2]_{total} - [H2]_{incorporated}$. The yellow trace in Figure 3 shows $[H2]_{incorporated}$ at the concentrations of molecules simulated. The simulated concentration of H2 is consistent with our measurements of H2 incorporation in the polymer. In the simulation, the number of insertion sites initially decreases, as the sites interact with Hairpin 1, and then the number of insertion sites increases exponentially fast until a large fraction of the hairpins are consumed and the system reaches an equilibrium. The growing number of insertion sites may serve as a proxy for the total concentration of polymer in our system, which we have not measured.

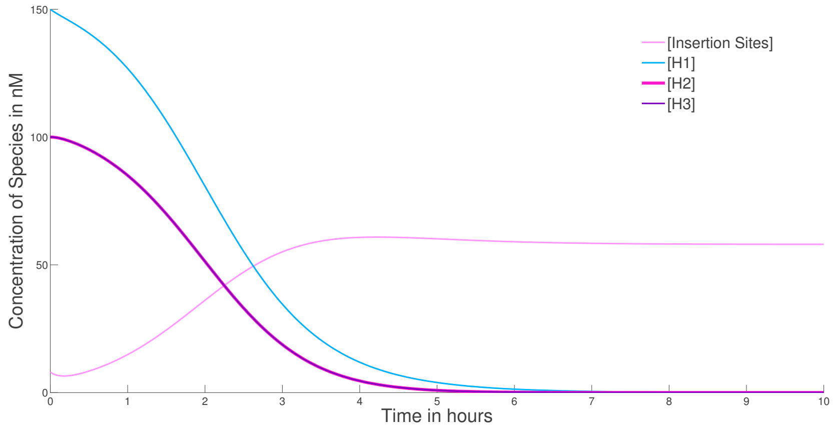


Figure S9: The ordinary differential equation model of the exponential growth system discussed in Section 4.5 is simulated. The number of insertion sites initially decreases, as the sites interact with Hairpin 1, and then the sites begin to increase exponentially fast until a large fraction of the hairpins are consumed and the system reaches an equilibrium.

# S3 Parameter Fitting

The rate at which the number of H1 insertion sites [I] increases is

$$\frac{d[I]}{dt} = k[I][H1]. \tag{23}$$

We examine this rate at $10\%$ completion time, because at that time the concentration of H1 is roughly constant, and the number of Insertion sites [I] is approximately $10\%$ of $[H1]_0$. We substitute $C$ for $k \times [H1]_0$ and $I(t_{10\%})$ for $0.1[H1]_0$ to get

$$\frac{d[I]}{dt} = C[I]. \tag{24}$$

Integrating this equation on both sides gives:

$$[I] = A \times e^{C \times t}, \tag{25}$$

where A is a constant determined by $[I]_0$. Thus

$$[I] = [I]_0 \times e^{C \times t}. \tag{26}$$

When t = 0, $[A] = [I]_0$. $C$ is a measure of how quickly the number of H1 insertion sites double ($\frac{1}{C}$ is the slope of the plot comparing relative Initiator concentration to $ln(t_{10\%})$). This value is derived as follows:

$$[I](t_{10\%}) = [I]_0 \times e^{(C \times t_{10\%})}, \tag{27}$$

and

$$0.1[H1]_0 = [I]_0 \times e^{(k \times [H1]_0 \times t_{10\%})}. \tag{28}$$

Now we can divide both sides by $[I]_0$ and take the natural log of both sides to get:

$$\ln(\frac{0.1[H1]_0}{I_0}) = (k \times [H1]_0 \times t_{10\%}). \tag{29}$$

We performed a linear fit on $\ln(\frac{0.1[H1]_0}{I_0})$ and the $10\%$ completion time on our spectrofluorimetry data (Figure 3). The slope of this line is $\frac{1}{C}$ or $\frac{1}{k \times [H1]_0}$ where $k$ is the reaction rate constant. The slope of

this line is 0.3667 hours or 1320 seconds. $[\text{H1}]_0$ in these experiments is 150 nM. Therefore:

$$k = \frac{1}{slope \times [H1]_0}, \tag{30}$$

$$k = \frac{1}{1320 \times 1.5 \times 10^{-7}}, \tag{31}$$

$$k = 5050 M^{-1} sec^{-1}. \tag{32}$$

This is the value used in our ordinary differential equation simulation discussed in Section 4.5.

# S4 Exponential Growth System Experiments

In the remainder of the supplementary section we include sufficient detail into our experiments such that the reader can gain an intuition into how our system works and can reproduce our work and if they so choose. We include detailed explanations of our control experiments, length measurement experiments, and time lapse experiments in this section as well as our polymer division experiment, our treadmilling polymer design and a more detailed exploration of the leak in our system in the sections that follow. Our molecular system utilizes the 4-way branch migration mechanism demonstrated in figure S10.

### DNA Sequences

All of the experimental results shown in this manuscript used the DNA sequences listed in Table S1, we refer to this design as “6-3v1” to refer to an toehold sizes of 6 and 3. Other design lengths and sequences were explored, but the alternate designs resulted in a larger system leaks than the sequences presented here (see Section S6).

### Control Experiments

We conducted the control experiments described in this section in order to verify that our system does indeed grow a linear polymer (as opposed to an amorphous blob of DNA) via the asynchronous insertion of hairpins after growth is triggered by an initiator.

Figure S11 shows a non-denaturing gel control that we used to show if there are any undesired interactions between reactants in our system. Every subset of reactants is tested at the same concentration 100 nM. Figure S11 shows that a small undesired leak occurs between reactants Hairpin 1 (H1) and Hairpin 2 (H2) and between reactants Hairpin 1 (H1) and Hairpin 3 (H3). However, no leak occurs between any of the other species. via spectrofluorimetry experiments in Figure 3. The leak is small enough that it does not interfere significantly with our experiments.

We also performed a control time-lapse gel to test the system for polymer joining (to see if the polymer can grow by smaller polymers randomly joining each other at the ends), see Figure S12. In this time lapse reaction of the experimental system we set the [Initiator] equivalent to hairpin concentrations. We tagged Hairpin 2 with a fluorophore and quencher pair, such that the unreacted Hairpin 2 is quenched, but when Hairpin2 is integrated into the polymer the hairpin is opened and the fluorophore is no longer quenched. A sketch of the experimental design is shown in Figure S13. The gel on the left and right of Figure S12 are the same but imaged under different conditions: the left image shows the gel prior to staining and imaged at the fluorophore emission wavelength; on the right is the same gel stained with SYBR Gold and imaged at the SYBR Gold emission wavelength. This experimental designed and imaging prior to staining allows us to visualize the gel bands where darkness of the band is commensurate with the number of Hairpin 2 molecules that

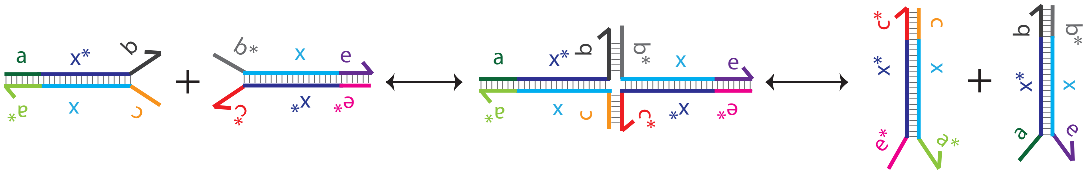

Figure S10: An illustration of the 4-way branch migration mechanism. The two monomers to the left can reversibly bind to each other and form a four-strand complex in the middle. The four-strand complex can reversibly split into the two monomers to the right. The rates of the 4-way branch migration can be controlled by the length of the toeholds $b, c, a, e$.

Table S1: 6-3 Toehold Design Version1

| | |
|---|---|
| m6n3 Insertion Init1.v1 | ACCGCACGTCCACGGTGTCGCACCCAC |
| m6n3 Insertion Init2.v1 | AACGCGACACCGTGGACGTGCGGT |
| m6n3 Insertion Init2.ROX | AACGCGACACCGTGGACGTGCGGT /3Rox_N/ |
| m6n3 Insertion_H1.v1 | GTGGGTGCGACACCGTGGACGTGCCTCAGACCAAGAGCACGTCCACGGTGTCGCGTT |
| m6n3 Insertion_H2.v1 | TCTGAGGCACGTCCACGGTGTCGCACCCACAACGCGACACCGTGGACGTGCGGT |
| m6n3 Insertion_H3.v1 | ACCGCACGTCCACGGTGTCGCACCCACAACGCGACACCGTGGACGTGCTCTTGG |
| m6n3 Divide_H1-3' | CCAAGAGCACGTCCACGGTGTCGCGTT |
| m6n3 Divide_H1-5' | GTGGGTGCGACACCGTGGACGTGCCTCAGA |
| m6n3v1.H2-Rox-Quench | /5IAbRQ/TCTGAGGCACGTCCACGGTGTCGCACCCACAACGCGACACCGTGGACGTGCGGT/3Rox_N/ |
| m6n3 Linear_H3.v1 | ACCGCACGTCCACGGTGTCGCTTTTTTTTTGCGACACCG TGG ACG TGC TCT TGG |
| m6n3v1.H2RQ_DISPLACE | ACCGCACGTCCACGG TGT CGC GTT GTG GGT GCG ACA CCG TGG ACG TGC CTC AGA |

have been incorporated into the polymer at that band length. We stain with Syber Gold to visualize the gel ladder and interpret the bands' mass. If the polymers are randomly joining we would see an upward shift in the gel bands over time. This data shows that there is minimal joining. Top and bottom halves of the gel were loaded in a horizontal gel bed prior to running electrical current, to ensure that each lane was assayed under the identical circumstances.

## Length measurement experiments

We conducted the polymer length experiments described herein to verify that the polymer grows in chunks that are approximately the length of the number of bases in the hairpins and that the length decreases in relation to an increase in initiator concentration. We conducted these experiments by tagging only the Initiator molecule so that we could distinguish the initiated polymers from the leak polymers.

The average length of the polymer shrinks with increasing Initiator concentration in both the linear and exponential systems. We used a similar exper-

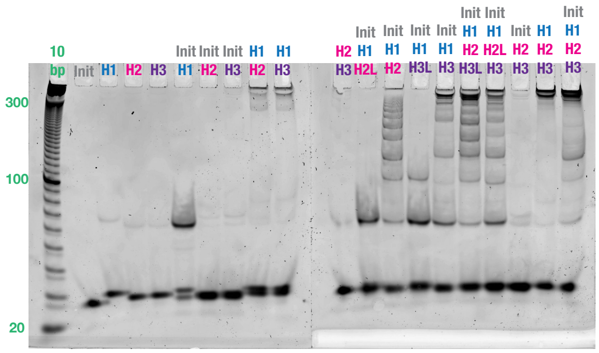


Figure S11: Combinatorial gel for 6-3v1 design. This is the exponential growth system described in Figure 1. Non-denaturing gel control experiments show if there are undesired interactions between reactants. The goal of non-denaturing gel control experiments (as shown above) is to show if there are undesired interactions between each combination of each reactant. The polyacrylamide gel above shows that a small undesired leak occurs between reactants Hairpin 1 (H1) and Hairpin 2 (H2) and between reactants Hairpin 1 (H1) and Hairpin 3 (H3). However, no leak occurs between any of the other species. We fit the leak via spectrofluorimetry experiments in Figure 3. The leak is small enough that it doesn't interfere significantly with our experiments. All species are present at 100 nM concentration.

imental scheme as that shown in Figure S13, with the fluorophore and quencher pair labeling the Initiator complex instead of Hairpin 2. When the Initiator is opened by it's interaction with Hairpin one. the quencher is moved away from the fluorophore and the new polymer can emit photons. The result is that each "Initiated polymer" is tagged exactly once, the leaked polymers are unlabeled by the fluorophore. We then tested the effect of varying the Initiator concentration on the length of the polymer. Figure S14 shows the Rox fluorescence intensity of the distribution of polymer lengths generated in the presence of Initiator concentrations $[I]_0 = \{0\%, 1\%, 2\%, 4\%, 8\%, 16\%, 32\%, 64\%, 100\%\}$ relative to hairpin concentrations after 6 hours of reaction with $1\mu$M concentrations of each hairpin in the linear (left) and exponential (right) systems. Lanes with Initiator concentrations smaller than 8% are difficult to resolve by eye in this gel as they contain less ROX-labeled Initiator. Figure S15 shows the same linear and exponential system gels after they have been stained by Syber Gold, so that we can measure the lengths of the polymers against a DNA ladder.

We are also able to observe quantized bands that approximate the length of the hairpins – 27 base-pairs.

Taken together (the flurescence tagged data from Figure S14, coupled with the polymer length extracted from the stained image of the gels in Figure S15 we get the normalized distributions of polymer size shown in Figure S16. This binned data shows a decrease in the mean length of polymer with increased Initiator concentration in both the linear and exponential systems. Note that we expect the binned values to be approximately the same in both the linear and exponential systems as the gel shows the result after 6 hours of reaction time, by which all of the hairpins in both systems are used up. The up-

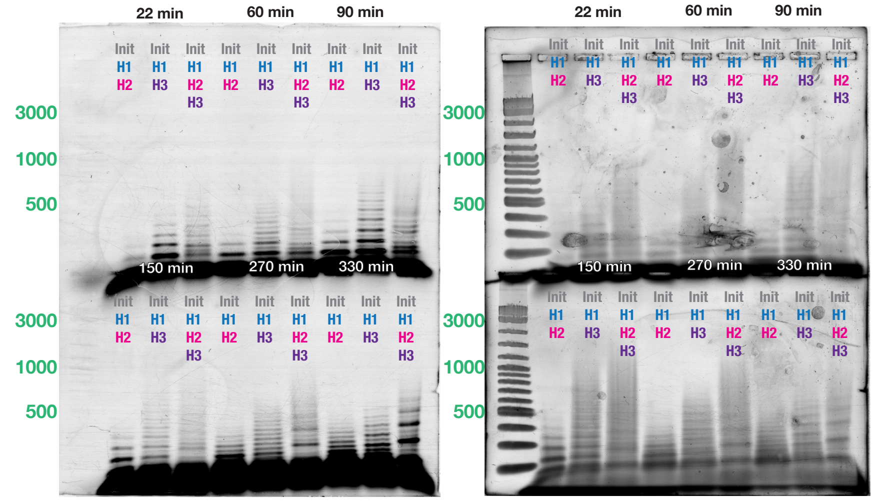


Figure S12: The time lapse reaction of the experimental system with [Initiator] equivalent to hairpin concentrations. The gel on the left and right are the same but imaged under different conditions (left no stain, imaged at fluorophore emission wavelength; on the right is the same gel stained with SYBR Gold and imaged at SYBR Gold emission wavelength). If the polymers are randomly joining we would see an upward shift in the gel bands over time. This data shows that there is minimal joining.

per limit of the y-axis for each distribution is 40% of the total concentration to allow the reader to clearly see the change in distribution. Vertical lines indicate the lower bound on the mean of the distribution, as calculated with all polymers larger than 800 base pairs being assigned to the bin of "$>$ 800" base pairs in length. The mean of each distribution in which more than 8% Initiator was utilized decreases with increasing amounts of Initiator.

Finally, we were able to visually confirm linear polymer growth and visualize the leak using Atomic Force Microscopy in Figure S17. Figure S17a shows a wide field image of the polymer product generated with the addition of 10% Initiator concentration relative to the other hairpins after 5 hours of reaction time(note that the scale bar in the image is 1 um). Figure S17b shows a zoomed in image of one of these polymers (note that the scale bar is 250 nm). Figure S17c, shows a wide field image of the leak formed by hairpins in the absence of Initiator (note that the scale bar is 1 um). And Figure S17d, shows a close up image of one of these leak products (the scale bar in this image is 250 nm).

## Time Lapse Experiments

In this subsection, we provide extra data on time lapse gels for linear and exponential growth systems. For the linear growth system, 80 nM ROX-labeled Initiator, and 1 $\mu$M of Hairpin 2 and Hairpin 3L are added initially. 1.5 $\mu$M Hairpin 1 is added at different time points (5-480 minutes prior to loading the gels). The system is expected to grow linearly after adding Hairpin 1. For the exponential growth system, 80 nM ROX-labeled Initiator, and 1 $\mu$M of Hairpin 2 and Hairpin 3 are added initially. 1.5 $\mu$M Hair-

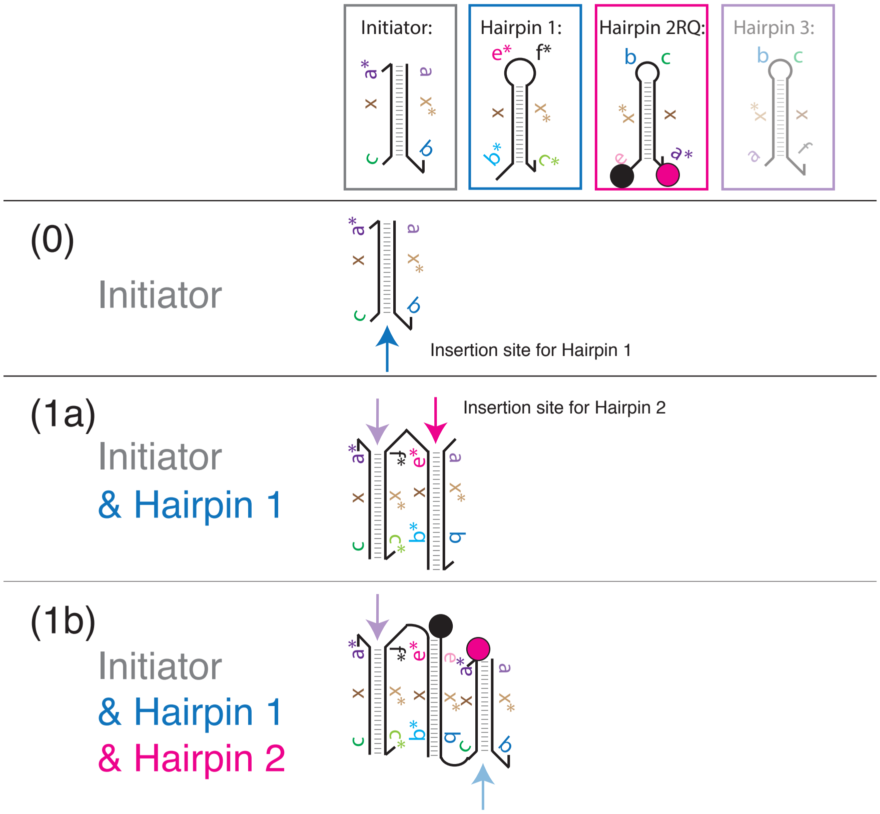


Figure S13: The experimental design for measuring conversion of monomer hairpins into the polymer. Above is a modified version of the schematic from Figure 1. The boxes around each oligonucleotide correspond to the insertion arrows in the mechanism below, which shows the incorporation of Hairpin 1 (1a) and Hairpin 2 (1b) into the polymer. Note that Hairpin 2RQ is a modified version of Hairpin 2 that includes a fluorophore-quencher pair. The fluorophore (pink circle) is quenched before Hairpin 2RQ reacts with the polymer. Upon Hairpin 2RQ's insertion into the polymer (1b), the fluorophore (pink circle) and quencher (black circle) are separated and the fluorophore emits light. We measure the kinetics of the incorporation of Hairpin 2RQ into the polymer by measuring the increase of fluorescence in tre he solution over time.

pin 1 is added at different time points (5-480 minutes prior to loading the gels). The system is expected to grow exponentially after adding Hairpin 1.

Figures 2A and 2B are the image of ROX fluorescence prior to staining with SYBR Gold for the linear and exponential growth systems respectively. The results for two extra experiments on the exponential system is in Figures S18 and S19. One similar experiment is in Figure S22. The result for the linear system after being stained with SYBR Gold is shown in Figure S20 (top). The results for the exponential system after being stained with SYBR Gold is shown in Figure S20 (bottom) and Figure S21.

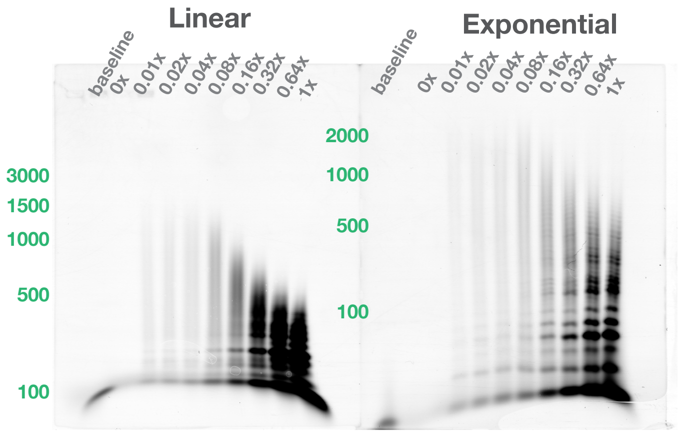


Figure S14: The average length of the polymer shrinks with increasing Initiator concentration (left to right) in both the linear and exponential systems. Rox fluorescence intensity imaged in this Super Fine Resolution Agarose gel shows the distribution of polymer lengths generated in the presence of Initiator concentrations $[I]_0 = \{0\%, 1\%, 2\%, 4\%, 8\%, 16\%, 32\%, 64\%, 100\%\}$ relative to hairpin concentrations after 6 hours of reaction with $1\mu$M Hairpins. Lanes with Initiator concentrations smaller than 8% are difficult to resolve by eye in this gel as they contain less ROX-labeled Initiator. Figure S15 shows an image of this gel after staining with SYBR Gold.

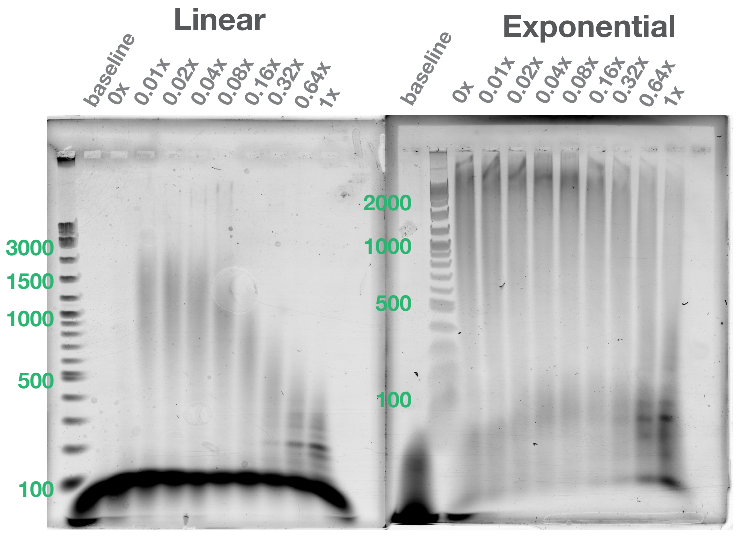


Figure S15: The Spectrofluorimetry linear and exponential final values gels from Figure S14 are shown here after being stained with SYBR Gold. Post-staining makes the DNA ladder visible, allowing for the proper size classification of the polymers.

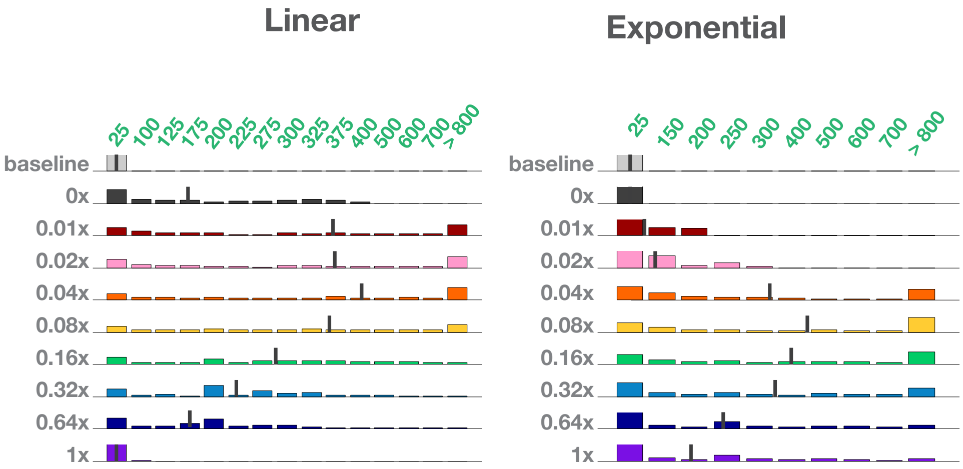


Figure S16: Normalized distributions of polymer length from the data in Figure S14 show a decrease in the mean length of polymer with increased Initiator concentration in both the linear and exponential systems. The upper limit of the y-axis for each distribution is 40% of the total concentration to allow the reader to clearly see the change in distribution. Vertical lines indicate the lower bound on the mean of the distribution, as calculated with all polymers larger than 800 base pairs being assigned a length of 800 base pairs. The mean of each distribution in which more than 8% Initiator was utilized decreases with increasing amounts of Initiator.

# S5 Division Experiments

We performed gel experiments to verify that the division complexes can divide our linear polymers into small fragments. The result indicate that most linear polymers are divided into small fractions even if we add the division complexes after the linear polymer have fully formed (six hours after the growth started).

In these division experiments, 80 nM ROX-labeled Initiator and 1 $\mu$M Hairpin 1, Hairpin 2, and Hairpin 3 are added initially. Divide complex are added at 0 and 6 hours respectively. The size of polymers decreases with increased concentrations of Divide complex. The results are shown in Figures S23 and S24. Figures S25 and S26 show the amount of polymers at different lengths in the above two gels.

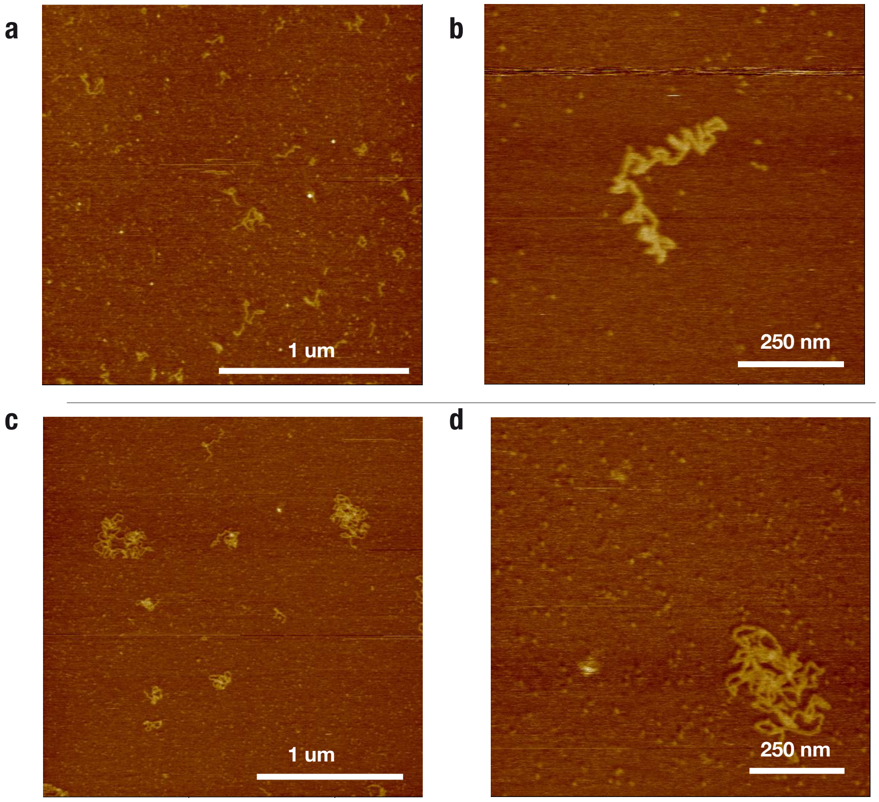


Figure S17: Atomic Force Microscopy images of exponentially grown polymer and leak product. a, Wide field image of polymer with 10% Initiator strand relative to the other hairpins after 5 hours (scale bar is 1 um). b, One of these polymers (scale bar is 250 nm). c, Wide field image of the leak formed by hairpins in the absence of Initiator (scale bar is 1 um). d, One of these leak products (scale bar is 250 nm).

## Division Kinetics

We performed kinetic experiments to test the speed of division. The Divide complex is added at the start of the growth. Figures S27 and S29 describe the results with 10% and 25% Divide complex respectively. The fluorescence signal describes the rate at which Hairpin 2 is incorporated into the growing and dividing polymer, which is the growth rate of all growing and dividing polymers combined. The flu-

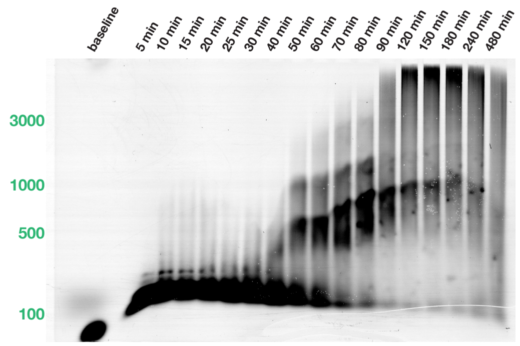


Figure S18: Gel time-lapse studies of exponential polymer growth. Super Fine Resolution Agarose non-denaturing gels of the product of a polymerization reaction with 80 nM ROX-labeled Initiator, 1.5 $\mu$M Hairpin 1, and 1 $\mu$M of Hairpin 2 and Hairpin 3. The system is expected to grow exponentially, as described in equations 18 to 22. ROX fluorescence was imaged prior to staining with SYBR Gold. Three additional experimental runs of this experiment can be found in Figures 2B, S19, and S22.

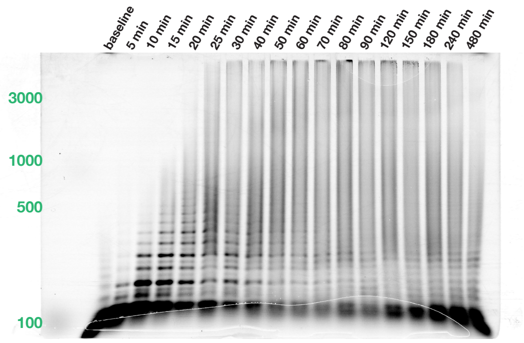


Figure S19: Gel time-lapse studies of exponential polymer growth. Super Fine Resolution Agarose non-denaturing gels of the product of a polymerization reaction with 80 nM ROX-labeled Initiator, 1.5 $\mu$M Hairpin 1, and 1 $\mu$M of Hairpin 2 and Hairpin 3. ROX fluorescence was imaged prior to staining with SYBR Gold. Three additional experimental runs of this experiment can be found in Figures 2B, S18 and S22.

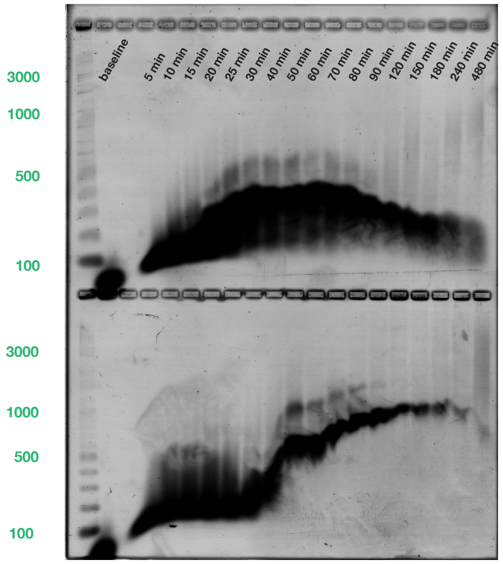


Figure S20: The time lapse gels from Figure 2A (top) and Figure S18 (bottom) are shown here after being stained with SYBR Gold. Post-staining makes the DNA ladder visible, allowing for the proper size classification of the polymers.

orescence result shows that the growth rate of these polymers does not change drastically with the presence of the Divide complex. Combining with the gel result, it also shows that the rate at which dividing happens is at least comparable with the growth rate of the polymers.

## Treadmilling

Figure S31 describes the design of the treadmilling system. The design requires a different set of monomers with structures different from the exponential growth systems. In this system, two monomers (Assembly 1 and Assembly 2) can insert alternatively near one end of the linear polymer while the other two monomers (Divide 1 and Divide 2) can cut the polymer near the other end. Notice

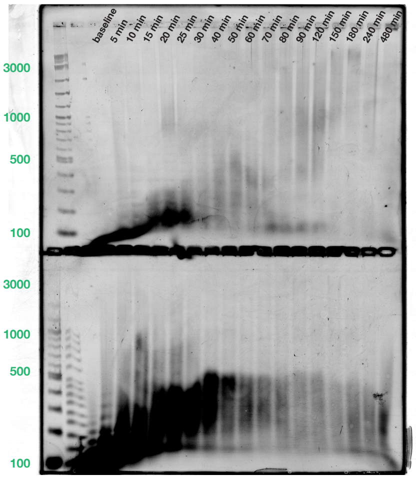


Figure S21: The time lapse gels from Figure 2B (top) and Figure S19 (bottom) are shown after being stained with SYBR Gold. Post-staining makes the DNA ladder visible, allowing for the proper size classification of the polymers.

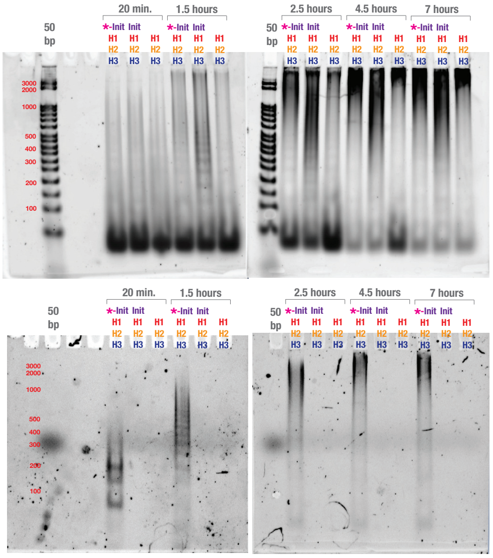


Figure S22: Gel time-lapse studies of polymer growth. a) Polyacrylamide gels of the product of a reaction with 50 nM Initiator, 625 nM Hairpin 1, and 500nM of Hairpin2 and Hairpin 3. b) Agarose gels of the same samples.

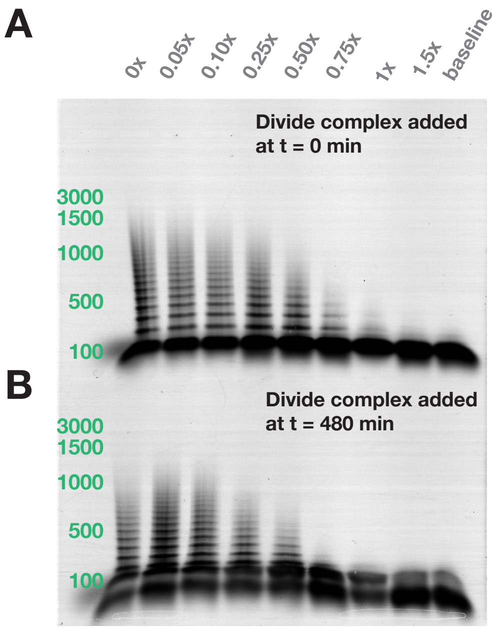


Figure S23: Polymer division. Super Fine Resolution Agarose non-denaturing gels of the product of a polymerization reaction with 80 nM ROX-labeled Initiator and 1 $\mu$M Hairpin 1, Hairpin 2, and Hairpin 3, to which Divide complex was added at concentrations $[D]_0 = \{0\%, 5\%, 10\%, 25\%, 50\%, 75\%, 100\%, 150\%\}$ relative to hairpin concentrations. (A) Divide complex was added with the hairpins at $t = 0$ min. (B) Divide complex was added after 6 hours of reaction. See Figure S23(B) for gel after staining with SYBR Gold.

that this is only an unimplemented theoretical design. The mechanisms used in this design are almost identical to those used in our exponential growth and division systems, even though the molecular structures are different. However, in order to demonstrate the treadmilling function, the reaction rates must be carefully chosen such that the growth rate is slightly larger than the division rates. This can be done by a careful selection of toehold lengths and sequences.

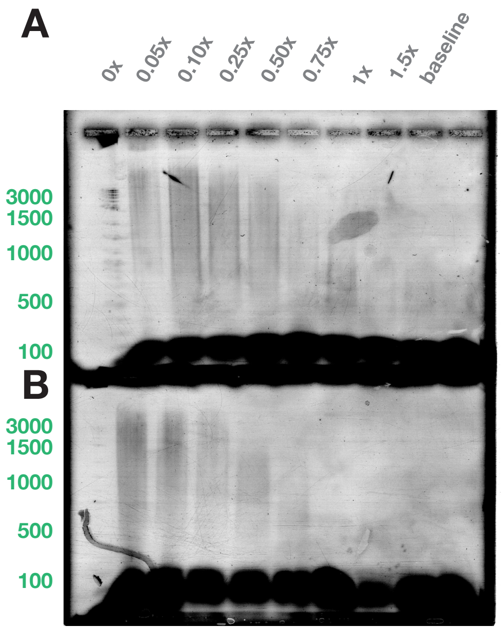


Figure S24: The polymer division gels from Figure S23 are shown here after being stained with SYBR Gold. Post-staining makes the DNA ladder visible, allowing for the proper size classification of the polymers.

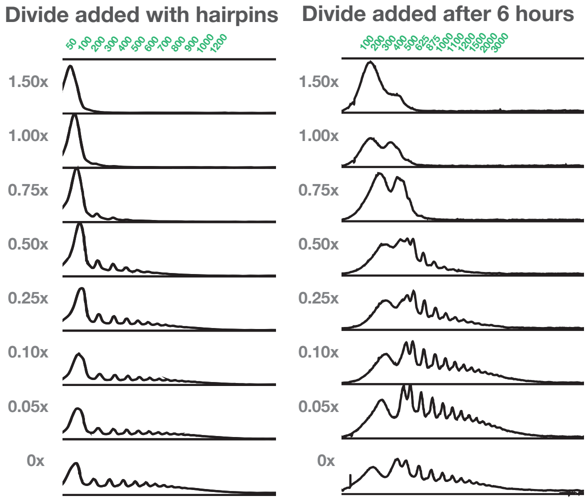


Figure S25: Division gel traces of the polymerization system with Divide complex added with hairpins (left) and six hours after hairpins are added (right) from Figure S23. Green numbers specify the size of each band, which are indicated as bumps in the gel traces.

## S6 Leakage in Various Designs

We modified toeholds $b$ and $c$ in the exponential growth system in three different ways and tested growth and leakage rates with gel experiments. The 6-3v2 design uses the same toehold length (6 and 3) but has a different sequence. The 5-4v2 design changes the toehold length to 5 and 4 respectively. As shown in Figures S34 and S33, in both the 6-3v2 and 5-4v2 designs, the growth rates are similar to our original design but the leak is larger. The 4-4v1 changes the toehold length to 4 and 4. As shown in Figure S32, the 4-4v1 design has significantly slower growth and leakage rates. We also performed a more extensive series of tests focusing on how the length of the toeholds ranging from 0 to 6 on both sides affect the reaction speed of the four-way branch migration. See chapter 5 of [5] for more details. Since it is not possible to test all sequence combinations, with the stem length of 18 bases, we made the assumption that the sequence of the stem affects the four-way branch migration mechanism less than the toehold lengths.

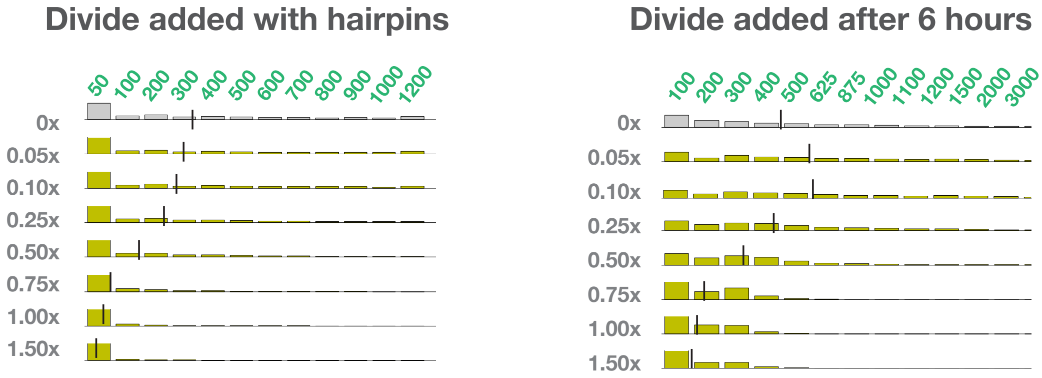


Figure S26: Division gel analysis of systems with Divide complex added with hairpins (left) and six hours after hairpins are added (right). The traces from Figure S25 were binned such that each bump in the trace was allotted to one bin. The y-axis for each distribution is 40% of the total concentration to allow the reader to clearly see the change in distribution. Vertical lines indicate the mean of the distribution. The mean of each distribution decreases with increased amounts of the Divide complex.

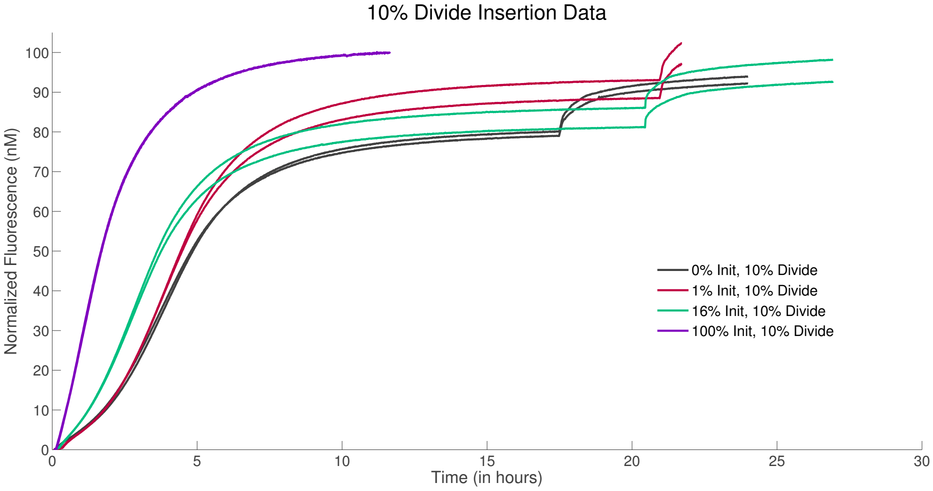


Figure S27: Polymer division kinetics examined via fluorescence. As Hairpin 2 is incorporated into the growing and dividing polymer, the system's fluorescence increases. Plotted above are the kinetic traces of Hairpin 2 (all hairpins are present at 100 nM, the Divide complex is present at 10 nM) with varying amounts of Initiator.

# References

[1] H. Abelson, D. Allen, D. Coore, C. Hanson, G. Homsy, T.F. Knight Jr, R. Nagpal, E. Rauch, G.J. Sussman, and R. Weiss. “Amorphous Computing”. *Communications of the ACM*,

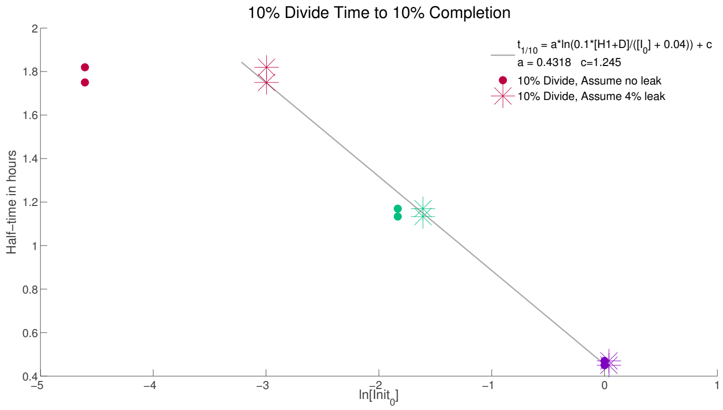


Figure S28: Linear fit of the $10\%$ completion time as a function of the relative concentration of Initiator to Hairpins. Filled circles correspond to a system where we assume no leak. Asterisks indicated the same points but assuming a leak equivalent to $4\%$ of the Initiator concentration.

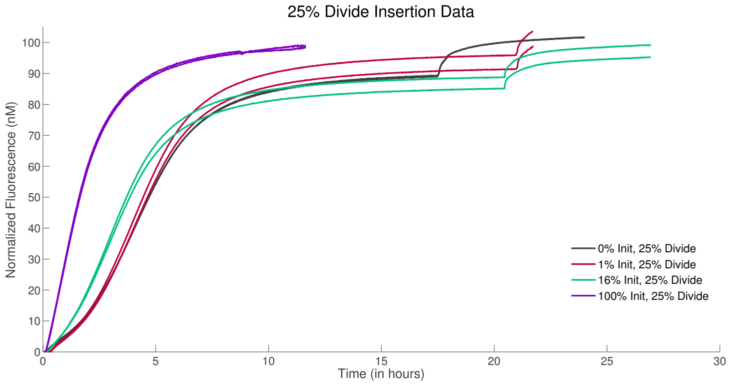


Figure S29: Polymer division kinetics examined via fluorescence. As Hairpin 2 is incorporated into the growing and dividing polymer, the system's fluorescence increases. Plotted above are the kinetic traces of Hairpin 2 (all hairpins are present at 100 nM, the Divide complex is present at 25 nM) with varying amounts of Initiator.

Table S2: 4-4 Toehold Design Version1

| | |
|---|---|
| m4n4 Insertion_init1 | GAGAGCACGTCCACGGTGTCGCACCC |
| m4n4 Insertion_init2 | ACAGGCGACACCGTGGACGTGCTCTC |
| m4n4 H1 | GGGTGCGACACCGTGGACGTGCCAGCCTCGGCACGTCCACGGTGTCGCCTGT |
| m4n4 H2 | GCTGGCACGTCCACGGTGTCGCACCCACAGGCGACACCGTGGACGTGCTCTC |
| m4n4 H3 | GAGAGCACGTCCACGGTGTCGCACCCACAGGCGACACCGTGGACGTGCCGAG |

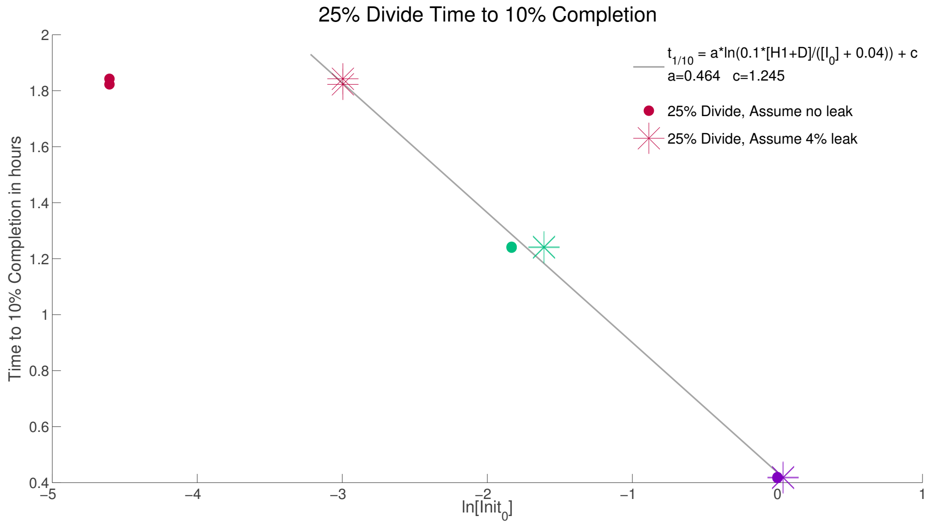


Figure S30: Linear fit of the $10\%$ completion time as a function of the relative concentration of Initiator to Hairpins. Filled circles correspond to a system where we assume no leak. Asterisks indicated the same points but assuming a leak equivalent to $4\%$ of the Initiator concentration.

Table S3: 5-4 Toehold Design Version2

| | |
|---|---|
| 5-4 v1.init1 | GTTA-GCCCTGTATTGGGCTCGC-TCTCG |
| 5-4 v1.init2 | GCCT-GCGAGCCCAATACAGGGC-TAAC |
| 5-4 v1.H1 | CGAGA-GCGAGCCCAATACAGGGC-ACTCA-ATCAC-GCCCTGTATTGGGCTCGC-AGGC |
| 5-4 v1.H2 | TGAGT-GCCCTGTATTGGGCTCGC-TCTCG-GCCT-GCGAGCCCAATACAGGGC-TAAC |
| 5-4 v1.H3 | GTTA-GCCCTGTATTGGGCTCGC-TCTCG-GCCT-GCGAGCCCAATACAGGGC-GTGAT |

Table S4: 6-3 Toehold Design Version 2

| | |
|---|---|
| 6-3v2.init1 | GTC-CGGGACGGACCCGTGCGC-CTTACG |
| 6-3v2.init2 | CTT-GCGCACGGGTCCGTCCCG-GAC |
| 6-3v2.H1 | CGTAAG-GCGCACGGGTCCGTCCCG-TGTCCA-AGCTAG-CGGGACGGACCCGTGCGC-AAG |
| 6-3v2.H2 | TGGACA-CGGGACGGACCCGTGCGC-CTTACG-CTT-GCGCACGGGTCCGTCCCG-GAC |
| 6-3v2.H3 | GTC-CGGGACGGACCCGTGCGC-CTTACG-CTT-GCGCACGGGTCCGTCCCG-CTAGCT |

43(5):74–82, 2000.

[2] L. Adleman. Molecular computation of solutions to combinatorial problems. *Science*, 266:1021–1024, 1994.

[3] Guy E. Blelloch. Programming parallel algorithms. *Commun. ACM*, 39(3):85–97, March

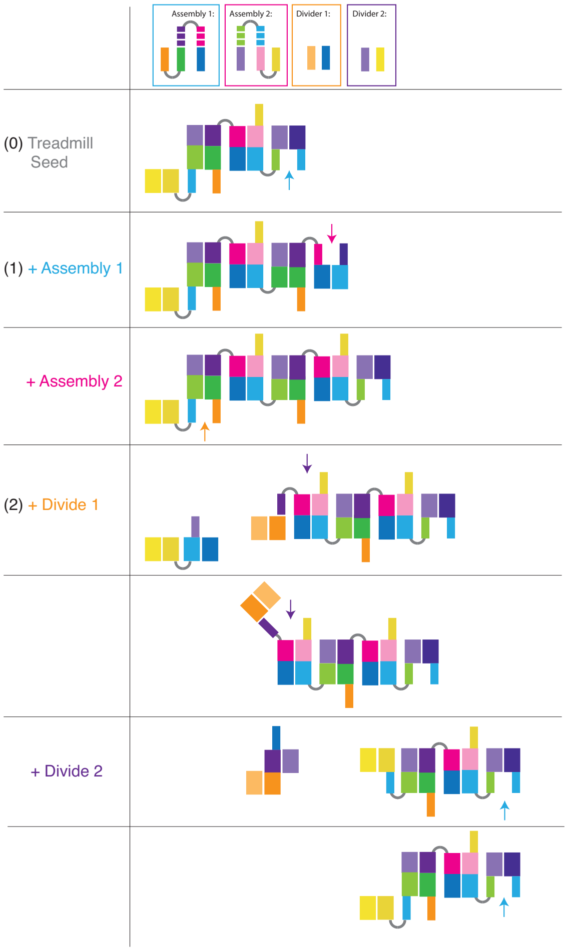


Figure S31: Combining insertion and division behaviors can result in treadmilling, the growth of one end of a polymer while the other end is shrinking. This design requires a different set of monomers and a different hairpin structure than what we have discussed and shown. While our treadmilling mechanism utilizes both insertion and division, the above design is incompatible with parallel insertion and division.


1996.

[4] Stephen A. Cook and Patrick W. Dymond. Parallel pointer machines. *Computational Complexity*, 3(1):19–30, Mar 1993.

[5] N Dabby. *Synthetic molecular machines for active self-assembly: prototype algorithms, designs, and experimental study*. PhD thesis, California Institute of Technology, 2013.

[6] Nadine Dabby, Alan Barr, and Ho-Lin Chen. “Part 1. Molecular System for an Exponentially Fast Growing Programmable Synthetic Polymer”. In submission to *Scientific Reports*, 2023.

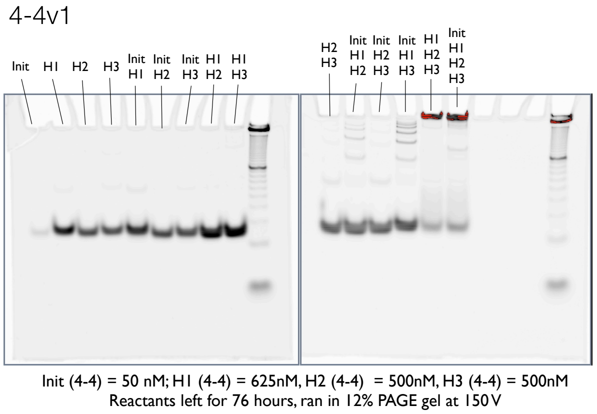


Figure S32: Combinatorial gel for 4-4v1 design. The polyacrylamide gel above shows that a small leak occurs between reactants Hairpin 1 (H1) and Hairpin 2 (H2) and Hairpin 3 (H3) in the absence of Initiator. This set of strands reacts significantly more slowly than the other designs.

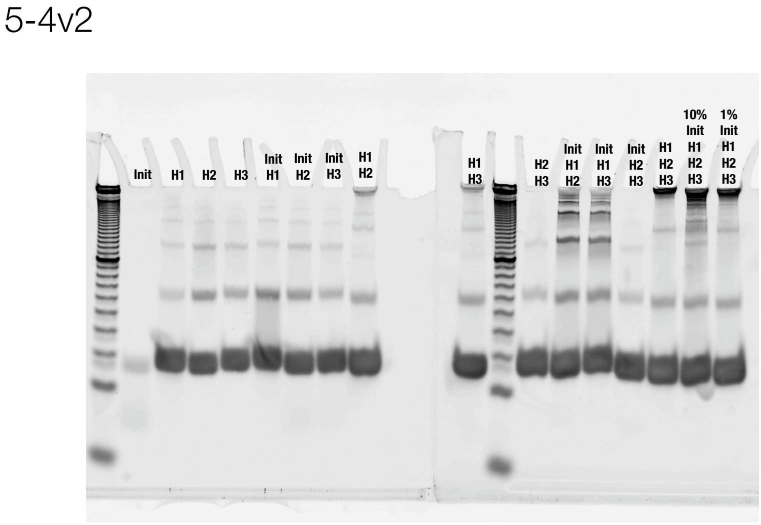


Figure S33: Combinatorial gel for 5-4v2 design. The polyacrylamide gel above shows that a small leak occurs between reactants Hairpin 1 (H1) and Hairpin 2 (H2) and between Hairpin 1 (H1) and Hairpin 3 (H3) in the absence of Initiator.

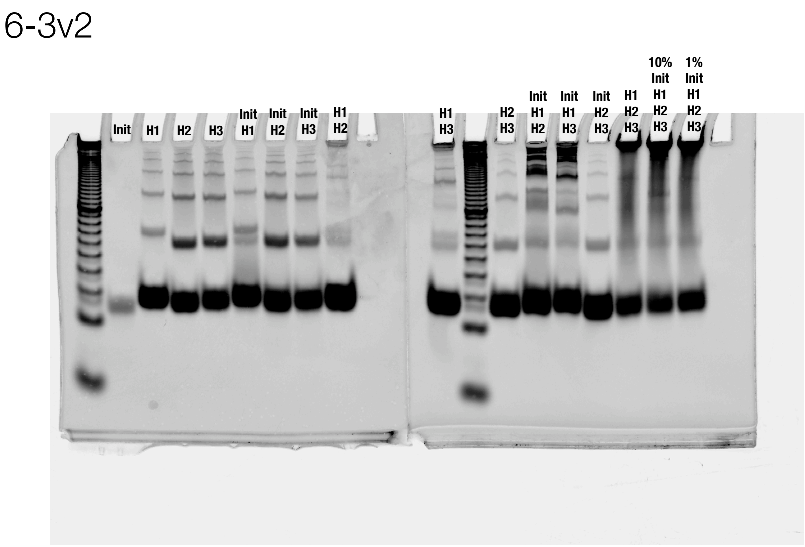


Figure S34: Combinatorial gel for 6-3v2 design. The polyacrylamide gel above shows that a small leak occurs between reactants Hairpin 1 (H1) and Hairpin 2 (H2) and between Hairpin 1 (H1) and Hairpin 3 (H3) in the absence of Initiator.

[7] Nadine Dabby and Ho-Lin Chen. “Active Self-Assembly of Simple Units Using an Insertion Primitive”. In *Proceedings of the Twenty-Fourth Annual ACM-SIAM Symposium on Discrete Algorithms*, pages 1526–1536. SIAM, 2013.

[8] M. Dale, S. Stepney, M. Trefzer, S. O’keefe, A. Sebald, and J. Dewhirst. The role of structure and complexity on reservoir computing quality. In *Proceedings of the International Conference on Unconventional Computation and Natural Computation*, pages 52–64, 2019.

[9] RP Feynman. Simulating physics with computers. *International Journal of Theoretical Physics*, 21(6-7):467–488, 1982.

[10] Benjamin Hescott, Caleb Malchik, and Andrew Winslow. ”Tight Bounds for Active Self-Assembly Using an Insertion Primitive”. *Algorithmica*, pages 1–18, 2015.

[11] W. Daniel Hillis and Guy L. Steele, Jr. Data parallel algorithms. *Commun. ACM*, 29(12):1170–1183, December 1986.

[12] N. Immerman. Expressibility and parallel complexity. *SIAM Journal on Computing*, 18(3):625–638, June 1989.

[13] E. Karsenti. “Self-Organization in Cell Biology: A Brief History”. *Nature Reviews Molecular Cell Biology*, 9(3):255–262, 2008.

[14] D. E. Knuth. *The Art of Computer Programming: Volume 3: Sorting and Searching*. Addison-Wesley, 1997.

[15] S Lloyd. Ultimate physical limits of computation. *Nature*, 406(6799):1047–1054, 2000.

[16] S. Stepney. Co-designing the computational model and the computing substrate. In *Proceedings of the International Conference on Unconventional Computation and Natural Computation*, pages 5–14, 2019.

[17] S. Stepney and V. Kendon. The role of the representational entity in physical computing. In *Proceedings of the International Conference on Unconventional Computation and Natural Computation*, pages 219–231, 2019.

[18] AM Turing. A reaction-diffusion model for development, the chemical basis of morphogenesis. *Phil Trans. Roy. Soc. of London, Series B*, pages 37–72, 1952.

[19] AM Turing. The chemical basis of morphogenesis. *Bulletin of Mathematical Biology*, 52(1-2):153–197, 1990.

[20] E. Winfree. "On the Computational Power of DNA Annealing and Ligation". In R.J. Lipton and E.B. Baum, editors, *DNA Based Computers*, pages 199–221. American Mathematical Society, Providence, RI, 1996.

[21] E. Winfree. *Algorithmic Self-Assembly of DNA*. PhD thesis, California Institute of Technology, Pasadena, 1998.

[22] D. Woods, H.-L. Chen, S. Goodfriend, N. Dabby, E. Winfree, and P. Yin. "Active Self-Assembly of Algorithmic Shapes and Patterns in Polylogarithmic Time". In *ITCS 2013: Innovations in Theoretical Computer Science*, pages 353–354, Berkeley, CA, January 2013. ACM.

[23] P. Yin, H.M.T. Choi, C.R. Calvert, and N.A. Pierce. "Programming Biomolecular Self-Assembly Pathways". *Nature*, 451(7176):318–322, 2008.
